# The kynurenine pathway in depression and schizophrenia: convergent signals, divergent states, and clinical signatures

**DOI:** 10.17179/excli2026-9522

**Published:** 2026-06-25

**Authors:** Masaru Tanaka, László Vécsei

**Affiliations:** 1 Danube Neuroscience Research Laboratory, HUN-REN-SZTE Neuroscience Research Group, Hungarian Research Network, University of Szeged, H-6725 Szeged, Hungary; 2 Department of Neurology, Albert Szent-Györgyi Medical School, University of Szeged, H-6725 Szeged, Hungary

**Keywords:** depressive disorder, major (MDD), depressive disorder, treatment-resistant (TRD), schizophrenia (SCZ), inflammation, kynurenine (KYN), biomarkers

## Abstract

Chronic low-grade inflammation (LGI) is increasingly recognized as a biologically meaningful contributor to heterogeneity in major psychiatric disorders. The tryptophan (Trp)-kynurenine (KYN) metabolic pathway is a leading candidate mechanism because immune and stress-related signals can redirect Trp metabolism toward bioactive KYNs that influence glutamatergic signaling, redox balance, energetics, and immune feedback. In treatment-resistant depression and schizophrenia spectrum psychosis, this pathway is especially relevant because inflammatory burden often coexists with anhedonia, fatigue, cognitive dysfunction, and negative symptoms. Yet the literature remains difficult to integrate. Studies often rely on shallow biomarker panels, inconsistent inflammatory phenotyping, mixed matrices, and incomplete handling of major confounders, including smoking, adiposity, sleep disruption, infection timing, and medication exposure. Interpretation is further complicated by the kynurenic acid (KYNA) paradox and by central-peripheral discrepancies, as KYNA-related findings are strongly shaped by biological context and compartment, with blood measures often diverging from cerebrospinal fluid profiles and therefore not reliably reflecting central branch balance. This review therefore aimed to identify the Trp-KYN nodes most relevant to chronic LGI in psychiatry, synthesize clinical and preclinical evidence by disorder and symptom module, and define realistic near- and long-term research priorities. Here we highlight that Trp-KYN findings become more coherent when interpreted as context-dependent branch-balance signatures rather than standalone biomarkers. This framework can improve comparability, sharpen stratification, and support biomarker-enriched translational psychiatry. More broadly, it offers a practical model for linking immune biology to symptom dimensions across heterogeneous brain disorders.

See also the graphical abstract(Fig. 1).

## Abbreviation List

AA, anthranilic acid; 3-HAA, 3-hydroxyanthranilic acid; 3-HK, 3-hydroxykynurenine; BMI, body mass index; CA, cinnabarinic acid; CRP, C-reactive protein; CSF, cerebrospinal fluid; HPA, hypothalamic-pituitary-adrenal; hsCRP, high-sensitivity C-reactive protein; IDO, indoleamine 2,3-dioxygenase; IDO1, indoleamine 2,3-dioxygenase 1; IDO2, indoleamine 2,3-dioxygenase 2; IFN, interferon; IL, interleukin; KAT, kynurenine aminotransferase; KMO, kynurenine 3-monooxygenase; KP, kynurenine pathway; KYN, kynurenine; KYN/Trp, kynurenine-to-tryptophan ratio; KYNA, kynurenic acid; LC-MS, liquid chromatography-tandem mass spectrometry; LGI, low-grade inflammation; NMDAR, N-methyl-D-aspartate receptor; NAD^+^, nicotinamide adenine dinucleotide; NMDA, N-methyl-D-aspartate; PA, picolinic acid; PBMC, peripheral blood mononuclear cell; PTSD, post-traumatic stress disorder; QA, quinolinic acid; QAA, quinaldic acid; QC, quality control; SCZ, schizophrenia; TDO, tryptophan 2,3-dioxygenase; TDO2, tryptophan 2,3-dioxygenase 2; TNF, tumor necrosis factor, TNF-α, tumor necrosis factor-alpha; TRD, treatment-resistant depression; Trp, tryptophan; XA, xanthurenic acid.

## 1. Introduction

Major depressive disorder, particularly treatment-resistant depression (TRD), and schizophrenia spectrum disorders remain leading drivers of disability, and their stubborn heterogeneity continues to frustrate biomarker discovery and treatment selection (Buch and Liston, 2021[64], Marquand et al., 2016[301], Serretti et al., 2025[433]). Across health systems and countries, resistance to standard treatments concentrates morbidity, suicidality, service use, and escalating direct and indirect costs, while psychosis similarly imposes enduring functional impairment and relapse-prone trajectories that erode recovery despite ongoing care (Gaynes et al., 2020[160], Siskind et al., 2022[438]). These realities sharpen the need for mechanistically grounded stratifiers that can explain why ostensibly similar patients diverge so dramatically in course and treatment response (Liloia et al., 2026[271], Marquand et al., 2016[301], Meehan et al., 2022[312], Solmi et al., 2023[442], Tanaka, 2025[465], Tanaka, 2025[466]). Among the candidate modifiers of this heterogeneity, immune activation and chronic low-grade inflammation (LGI) have emerged as repeat signals, albeit unevenly across patients (Goldsmith et al., 2016[167], Osimo et al., 2020[363], Yuan et al., 2019[534]).

Inflammatory phenotypes are not a blanket feature of psychiatric illness; they tend to concentrate in subgroups shaped by metabolic burden, smoking, sleep disruption, sedentary lifestyle, early-life adversity, and persistent psychosocial stress, factors that are overrepresented in both TRD and psychosis populations (Bauer and Teixeira, 2019[38], Byrne et al., 2022[68], Hassamal, 2023[189]). Frameworks for an inflammatory or immuno-metabolic subtype of depression converge on the idea that elevated high-sensitivity C-reactive protein (hsCRP) and cytokine signals co-occur with energy-related symptoms and poorer antidepressant response, making enrichment clinically feasible rather than theoretical (Figueiredo Godoy et al., 2025[140], Milaneschi et al., 2020[324], Miller, 2025[325], Zwiep et al., 2025[548]). Pragmatic stratifiers such as hsCRP cut-offs, atypical energy symptom scores, and multi-omic or imaging-informed clustering can therefore be used to define trial-ready strata in difficult-to-treat cohorts, with the explicit goal of increasing effect sizes and interpretability (Battaglia and Tanaka, 2026[37], Miller et al., 2025[326], Miller and Raison, 2023[327], Penninx et al., 2025[383], Tanaka, 2025[462], Tanaka, 2025[463]). This raises a translational challenge: we need mechanisms that can convert a high-inflammatory context into specific, testable neurochemical and symptom-level predictions (Goldsmith et al., 2023[166], Miller and Raison, 2023[327]). One reason this translation remains difficult is that the pathway's entry signals are rarely decomposed into their inflammatory and endocrine components (Maes et al., 2011[291], Tanaka et al., 2021[475], Tsuji et al., 2023[485]). In psychiatric cohorts, interpreting cortisol-linked tryptophan 2,3-dioxygenase (TDO) plausibility alongside cytokine-linked indoleamine 2,3-dioxygenase (IDO) plausibility may help explain why superficially similar tryptophan (Trp)-kynurenine (KYN) readouts diverge across diagnoses and clinical states (Fellendorf et al., 2022[138], Maes et al., 2011[291], Messaoud et al., 2019[317]). 

The Trp-KYN metabolic pathway is a compelling candidate for this role because immune signaling can reroute Trp metabolism toward KYNs that are not merely by-products, but bioactive mediators with immune and neurobiological effects (Badawy, 2017[27], Savitz, 2020[423], Tanaka and Battaglia, 2025[468], Tanaka et al., 2021[475]). This is mechanistically plausible because several KYNs engage receptor and redox-sensitive pathways, including N-methyl-D-aspartate receptor (NMDAR)-linked signaling, α7-nicotinic modulation, and NAD^+^-relevant energetics, and because immune cues can bias flux at multiple Trp-KYN nodes (Badawy, 2017[27], Juhász et al., 2025[219], Pathak et al., 2024[377], Stone et al., 2013[448]). Plausibility alone, however, is not evidence of in vivo pathway dominance in psychiatric cohorts, especially when panels stop at Trp, KYN, and KYN/Trp ratio (Almulla et al., 2022[14], Tanaka et al., 2021[475]). Psychiatric syntheses and cerebrospinal fluid (CSF)-focused studies further suggest that the direction of change is not uniform across disorders or compartments, reinforcing the need for branch-resolved interpretation rather than single-marker narratives (Huang et al., 2023[201], Lovelace et al., 2017[281], Tanaka et al., 2021[475]). If Trp-KYN is the switchboard, then the key question becomes which “switches” matter most-entry enzymes, branch points, or downstream effectors-and under what inflammatory conditions (Pires et al., 2022[388], Stone and Williams, 2024[450], Yan et al., 2024[522]). 

At the pathway's entry, inducible enzymes such as IDOs and stress-linked TDO govern KYN availability, while downstream branching, often conceptualized as a kynurenine aminotransferases (KATs) and kynurenic acid (KYNA) versus KMO and quinolinic acid (QA) tilt, can shape glutamatergic signaling, oxidative balance, and neuroimmune feedback loops (Badawy, 2017[27], Parrott and O'Connor, 2015[374], Sathyasaikumar et al., 2022[420], Stone and Williams, 2024[450]). Cell-type localization sharpens this logic: microglial kynurenine 3-monooxygenase (KMO) activity favors 3-hydroxykynurenine (3-HK) and QA with pro-oxidant and NMDAR agonist properties, whereas astrocytic KAT activity enriches KYNA with NMDAR and α7-nicotinic receptor antagonism (Anderson et al., 2021[20], Garrison et al., 2018[158], Parrott and O'Connor, 2015[374], Tanaka, 2026[464]). In TRD, a QA-leaning pattern is mechanistically plausible as a contributor to anhedonia and cognitive slowing because QA can amplify NMDAR-linked and pro-oxidant cascades, but plausibility alone is not evidence of in vivo pathway dominance in psychiatric cohorts (Anderson et al., 2021[20], Bansal et al., 2022[33], Hestad et al., 2022[192]). Mechanistic support is stronger when node perturbation or target engagement shifts a prespecified metabolite pattern and that change tracks a symptom module, ideally under defined immune context and with branch-resolving markers (Bai et al., 2021[32], Garrison et al., 2018[158], Zeng et al., 2025[537]). These downstream consequences align with the symptom dimensions that most resist treatment in TRD and psychosis, anhedonia, fatigue, cognitive dysfunction, and negative symptoms, suggesting a pathway-level lens may outperform diagnosis-only approaches (Aleman et al., 2017[9], Anderson et al., 2021[20], Hestad et al., 2022[192], Liloia et al., 2026[271], Tanaka et al., 2025[472]). 

Yet Trp-KYN findings in TRD and psychosis often appear inconsistent, largely because studies differ in biomarker depth (e.g., Trp/KYN ratios without downstream metabolites), biospecimens (serum vs plasma vs CSF), sampling conditions, and confounding structures such as smoking, adiposity, infection timing, and psychotropic exposure (Almulla et al., 2022[15], Marx et al., 2021[305]). Critical appraisals of blood quantification highlight preanalytic fragility, including fasting status, tube type, albumin binding, and free versus total fractions, while liquid chromatography-mass spectrometry (LC-MS) method reviews show wide variability in validation and cross-matrix coverage (Badawy and Guillemin, 2019[30]). Even normative datasets demonstrate that matrix and platform shift “baseline” Trp and KYN values, destabilizing cut-offs (Metri et al., 2023[319]). A coherent framework therefore requires integrating mechanism with measurement; otherwise, “differences” may reflect methodology rather than biology (Badawy and Guillemin, 2019[30]).

This review focuses on psychiatric disorders, emphasizing TRD and schizophrenia spectrum psychosis, and on transdiagnostic symptom modules within these conditions, while excluding studies where neurodegenerative or primary neurologic diseases form the main clinical context. Psychiatric Trp-KYN syntheses and meta-analyses already show rich, diagnosis- and compartment-sensitive Trp-KYN patterns that warrant a dedicated appraisal without being diluted by Alzheimer's, Parkinson's, or other neurologic primaries (Almulla et al., 2022[14], Inam et al., 2023[205]). When neurologic insults are discussed, they are considered only insofar as they illuminate secondary psychiatric outcomes (Chen et al., 2014[83], Taquet et al., 2021[478]). Within this boundary, the priority is to clarify what is known, what is uncertain, and what is missing (Inam et al., 2023[205], Muneer, 2020[343]). 

Recent meta-analyses and systematic reviews already establish several anchor points for the field (Arnone et al., 2018[22], Marx et al., 2021[305]). Across mood and psychosis disorders, the KYN pathway shows reproducible but non-uniform abnormalities, with stronger convergence for reduced Trp and context-sensitive shifts in KYN metabolites than for any single diagnosis-wide signature (Brum et al., 2023[62], Haroon et al., 2020[184], Liloia et al., 2026[271], Marx et al., 2021[305]). Meta-analytic work in depression, bipolar disorder, and SCZ also suggests that interpretation changes with compartment and panel depth: CSF studies more often support central branch imbalance, whereas blood-based studies are more heterogeneous and more vulnerable to matrix, treatment, and metabolic confounding (Inam et al., 2023[205], Skorobogatov et al., 2021[439]). Disorder-focused reviews further indicate that cognitive and reward-related phenotypes, as well as symptom dimensions linked to inflammation, may be more informative than diagnosis totals alone (Goldsmith et al., 2023[166], Kindler et al., 2020[237]). Taken together, these syntheses support the relevance of Trp-KYN biology to psychiatry, but they also show that shallow panels, inconsistent inflammatory phenotyping, and weak cross-compartment validation remain major barriers to integration (Haroon et al., 2020[184], Hunt et al., 2020[203], Skorobogatov et al., 2021[439]). The purpose of the present review is therefore not to revisit whether the pathway matters at all, but to clarify which nodes, contexts, and symptom-linked signals are most interpretable under chronic low-grade inflammatory conditions (Table 1(Tab. 1); References in Table 1: Almulla et al., 2022[14]; Bartoli et al., 2021[36]; Hunt et al., 2020[203]; Inam et al., 2023[205]; Marx et al., 2021[305]; Ogyu et al., 2018[355]; Sapienza et al., 2023[418]).

Accordingly, this narrative review addresses four questions designed to translate a broad pathway literature into an actionable framework for TRD and psychosis while retaining relevance across psychiatric disorders. First, which nodes of Trp-KYN metabolism are most relevant to chronic LGI in psychiatric disorders? Second, what does clinical evidence show by disorder and by symptom modules, particularly anhedonia, fatigue, cognition, and negative symptoms? Third, which preclinical data provide causal leverage linking immune perturbation, Trp-KYN shifts, and psychiatric-relevant behaviors? Fourth, what are the key gaps and the most realistic near- and long-term research directions for biomarkers and interventions? To answer these questions, we use a pathway-first structure that moves from biology to measurement and then to human and model evidence (Table 1(Tab. 1)). 

We begin by outlining Trp-KYN pathway architecture and its immune triggers, highlighting entry enzymes and branch points most likely to mediate low-grade inflammatory effects. We then discuss how the pathway is measured and interpreted, before synthesizing clinical findings with emphasis on TRD and psychosis and organizing outcomes by symptom modules alongside diagnoses. Next, we use mechanistic and translational frameworks that treat KYNs as context-dependent signals to reconcile apparent contradictions across matrices and cohorts, and we draw on emerging gut-brain and lifestyle-linked Trp-KYN models as integrative test beds. Finally, we integrate preclinical causal evidence to identify where translation is strongest, and we conclude by prioritizing the most urgent research gaps and a near- and long-term roadmap toward stratified psychiatry (Table 1(Tab. 1)). 

## 2. The Tryptophan–Kynurenine Metabolic Pathway as a Neuroimmune Switchboard

**Figure 2:**
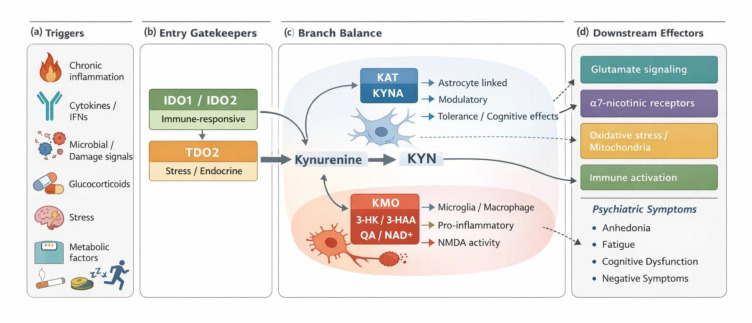
The Trp-KYN neuroimmune switchboard: enzymatic gatekeepers, branch asymmetry, and effector pathways shaping psychiatric phenotypes. Schematic overview of the Trp-KYN pathway as a neuroimmune decision network. Immune, endocrine, microbial, stress-related, and lifestyle cues converge on IDO1/IDO2 and TDO2, directing Trp toward KYN. KYN is then partitioned between a KAT isoform-conditioned KYNA arm, associated with astrocytic modulation and context-dependent neuroprotection, and a KMO-3-HK/3-HAA-QA/NAD^+^ arm, linked to microglial inflammatory signaling, redox stress, and NMDAR-related effects. These branch dynamics shape glutamatergic and α7-nicotinic signaling, mitochondrial function, immune feedback, and symptom domains including anhedonia, fatigue, cognitive dysfunction, and negative symptoms. 3-HAA, 3-hydroxyanthranilic acid; 3-HK, 3-hydroxykynurenine; α7nAChR, alpha-7 nicotinic acetylcholine receptor; IDO1, indoleamine 2,3-dioxygenase 1; IDO2, indoleamine 2,3-dioxygenase 2; KAT, kynurenine aminotransferase; KMO, kynurenine 3-monooxygenase; KYN, kynurenine; KYNA, kynurenic acid; NAD^+^, nicotinamide adenine dinucleotide; NMDA, N-methyl-D-aspartate; QA, quinolinic acid; TDO2, tryptophan 2,3-dioxygenase 2; Trp, tryptophan.

The Trp-KYN pathway is best understood not as a single linear cascade, but as a neuroimmune switchboard in which inflammatory and stress-related inputs regulate the entry of Trp into KYN metabolism, while downstream branching determines which bioactive metabolites dominate the chemical environment relevant to brain function and behavior (Savitz, 2020[423], Stone and Williams, 2024[450], Tsuji et al., 2023[485]). In this context-conditioned view, upstream gatekeepers such as cytokine-inducible IDOs and glucocorticoid-responsive TDO shape the rate of KYN production, whereas tissue- and cell-specific enzyme expression steers flux toward metabolites with distinct receptor, redox, and immunomodulatory properties (Stone and Williams, 2024[450], Tanaka and Vécsei, 2025[476], Tsuji et al., 2023[485]). The pathway therefore behaves less like a single biomarker axis and more like a routed signaling network whose outputs vary according to immune tone, endocrine state, biological compartment, and time scale (Savitz, 2020[423], Stone and Williams, 2024[450], Tsuji et al., 2023[485]). This section follows that logic from the entry gatekeepers to the principal branch points, and then to the downstream effector systems most often invoked in psychiatric phenotypes (Savitz, 2020[423], Stone and Williams, 2024[450]) (Figure 2(Fig. 2)). 

A key caveat is that this switchboard is distributed rather than unitary. Its outputs depend both on where flux is generated and on where it is measured, whether in liver, immune cells, endothelium, glia, blood, CSF, or ex vivo immune-cell systems (Savitz, 2020[423], Tanaka et al., 2021[475], Tsuji et al., 2023[485]). As a result, the same headline readout, particularly KYN/Trp ratio, can reflect different upstream drivers across cohorts and rarely resolves branch routing without deeper metabolite coverage (Krupa and Kowalska, 2021[244], Tanaka et al., 2021[475]). Throughout this section, three linked but distinct levels of inference are therefore kept separate: a biochemical claim that immune and stress-related signals alter Trp catabolism and KYN availability; a systems claim that branch dominance shapes glutamatergic, redox, and immune signaling; and a clinical claim that, under defined inflammatory contexts, these pathway shifts may track symptom modules or treatment-relevant phenotypes (Krupa and Kowalska, 2021[244], Savitz, 2020[423], Stone and Williams, 2024[450]). Keeping these levels apart matters because much of the apparent inconsistency in the literature arises when a context-dependent proxy is treated as if it were a complete biological explanation (Krupa and Kowalska, 2021[244], Tanaka et al., 2021[475]). 

### 2.1 Gatekeepers and triggers indoleamine 2,3-dioxygenases vs tryptophan 2,3-dioxygenase

At the entry point to KYN production, the main gatekeepers are the IDO isoforms and TDO (Badawy, 2017[27], Fatokun et al., 2013[132], Zhai et al., 2015[539]). These enzymes are often discussed together because they all catalyze the first step of Trp degradation, but they should not be treated as functionally interchangeable (Platten et al., 2019[389], Wu et al., 2018[516], Ye et al., 2019[531]). Among the IDO isoforms, IDO1 currently has the clearer evidence base as an inflammation-responsive driver of Trp-to-KYN conversion, whereas IDO2 remains less well characterized, more context-dependent, and less directly integrated into psychiatric biomarker interpretation (Krupa and Kowalska, 2021[244], Mondanelli et al., 2021[332], Pallotta et al., 2022[368], Zhai et al., 2015[539] ). For that reason, this review treats IDO1 as the principal immune-inducible entry enzyme while retaining IDO2 as a potentially relevant but less resolved modifier of entry control.

In broad terms, IDO-weighted regulation is most evident in immune-competent and barrier-related compartments, whereas TDO-weighted regulation is more closely tied to hepatic, metabolic, and glucocorticoid-linked physiology (Huang et al., 2022[200], Platten et al., 2019[389], Zhai et al., 2015[539]). The distinction is not absolute; both programs can appear outside their classical settings, and mixed states are likely common (Badawy and Guillemin, 2019[30], Wang et al., 2015[505], Ye et al., 2019[531]). Even so, separating them conceptually is useful because it helps distinguish immune-driven induction from stress-endocrine or metabolic shifts that may produce superficially similar changes in circulating KYN or KYN/Trp ratio (Badawy and Guillemin, 2019[30], Stone and Williams, 2023[449]). 

IDO1 is especially relevant to the neuroimmune framing of the pathway because it is commonly embedded within interferon (IFN)-related and broader inflammatory transcriptional programs that accelerate the conversion of Trp to KYN (Badawy, 2023[28], Strasser et al., 2017[451]). Across infection, sterile inflammatory states, and immune therapies, this pattern often appears as declining Trp, rising KYN, and higher KYN/Trp ratio, sometimes alongside parallel immune markers such as neopterin (Lanser et al., 2020[255], Strasser et al., 2017[451], Zhai et al., 2020[538]). Mechanistically, this matters for more than substrate depletion alone. Trp depletion and KYN accumulation can jointly contribute to tolerance-linked signaling, helping explain why inflammatory activation is repeatedly associated with fatigue, motivational disturbance, and depressive symptom burden (Chaves Filho et al., 2018[79], Lanser et al., 2020[255], Savonije et al., 2023[424]). At the same time, a plasma or serum increase in KYN/Trp ratio should not be overread. Identical ratios can arise from different tissue sources, and an immune-like profile at the level of entry control does not by itself establish whether downstream flux is being directed toward KYNA, toward KMO-linked products such as 3-HK and QA, or toward a mixed pattern (Larkin et al., 2016[256], Lu et al., 2025[282], Tanaka and Vécsei, 2025[476]). This caution is especially relevant because many studies infer “IDO activity” from KYN/Trp ratio or related entry indices without distinguishing whether the signal is most plausibly attributable to IDO1, IDO2, TDO, or a blended state (Meireson et al., 2020[315], Mor et al., 2024[335], Platten et al., 2019[389]).

A practical way to sharpen this inference in psychiatric cohorts is to pair pathway-entry indices with upstream endocrine and inflammatory context rather than reading KYN/Trp ratio in isolation (Haroon et al., 2020[184], Harris et al., 2024[185], Savitz, 2020[423]). At the diagnosis or phenotype level, higher cortisol provides a more plausible rationale for relative TDO weighting, whereas elevated CRP and pro-inflammatory cytokines provide a more plausible rationale for relative IDO weighting; when both are elevated, a mixed TDO/IDO state is usually the more defensible interpretation than a single-enzyme model (Badawy, 2017[27], Höglund et al., 2019[195], Messaoud et al., 2019[317]). Framed this way, the question shifts from whether a given cohort shows “IDO activation” in the abstract to whether its broader biomarker context is more consistent with glucocorticoid-linked, cytokine-linked, or convergent upstream pressure on Trp catabolism (Haroon et al., 2020[184], Savitz, 2020[423], Tanaka et al., 2021[475]). 

This distinction is especially useful in psychiatry because the balance between cortisol and inflammatory signaling appears to vary systematically across diagnoses and subtypes rather than collapsing into one uniform pattern (Goldsmith et al., 2023[166], Van Den Noortgate et al., 2025[494]). Melancholic, psychotic, or otherwise severe major depression and acute bipolar states are more plausibly read as mixed TDO/IDO phenotypes, whereas post-traumatic stress disorder (PTSD) more often resembles an inflammatory-dominant, relatively IDO-leaning state marked by blunted cortisol alongside elevated inflammatory markers; schizophrenia and first-episode psychosis often fall between these poles, with mixed or inflammation-leaning profiles shaped by acuity, psychosocial stress, and treatment exposure (Almulla et al., 2022[15], Olff and van Zuiden, 2017[357], Sarapultsev et al., 2020[419]). These patterns should still be presented as biologically informed inference rather than direct proof of enzyme activity, but they provide a more clinically legible framework for interpreting heterogeneous entry-level Trp-KYN findings across psychiatric populations (Farcas et al., 2023[128], Van Den Noortgate et al., 2025[494]). 

TDO links KYN production to endocrine and stress-related physiology. Glucocorticoid signaling and chronic stress are often discussed as drivers of increased Trp catabolism even in the absence of an overt inflammatory spike, providing a plausible route by which sustained hypothalamic-pituitary-adrenal (HPA) axis load can influence baseline KYN availability (Badawy, 2017[27], Savitz, 2020[423], Tanaka et al., 2021[475]). This perspective is particularly relevant in psychiatric settings where chronic stress biology, disrupted sleep, metabolic strain, and LGI may coexist (Harris et al., 2024[185], Jamshed et al., 2022[208], Savitz, 2020[423]). In those circumstances, the pathway may not reflect a clean “immune” or “endocrine” signal at all, but rather a blended state in which TDO-weighted and IDO-weighted inputs interact (Deng et al., 2021[109], Karu et al., 2016[227], Li et al., 2022[265]). That is one reason why KYN/Trp ratio is useful as an entry-level index of increased Trp catabolism, yet insufficient as a standalone mechanistic marker (Badawy and Guillemin, 2019[30], Hestad et al., 2022[192], Karu et al., 2016[227]). Once KYN has been generated, the decisive question becomes not simply how much is produced, but where that substrate is directed at the branch points downstream (Figure 2(Fig. 2)) (Almulla et al., 2022[15], Badawy, 2017[27], Deng et al., 2021[109], Messaoud et al., 2019[317]). 

### 2.2 Branching logic: kynurenine aminotransferase / kynurenic acid versus kynurenine 3-monooxygenase/quinolinic acid (and why balance beats single metabolites)

Once KYN metabolite production is engaged, the central systems-level issue becomes branch routing. KYN can be directed toward a KYNA-facing route, largely shaped by KATs, or toward a KMO-linked route that feeds 3-HK, 3-hydroxyanthranilic acid (3-HAA), QA, and de novo nicotinamide adenine dinucleotide (NAD^+^) chemistry (Auyeung et al., 2023[25], Song et al., 2017[446], Yang et al., 2024[527]). This branch asymmetry is more informative than KYN alone because the two trajectories differ in receptor engagement, redox implications, and links to immune activation (Joisten et al., 2021[214], Pires et al., 2022[388], Stone et al., 2013[448]). A high KYN value can coexist with either a KYNA-leaning or a QA-leaning profile, so single-metabolite interpretation is often biologically shallow (Joisten et al., 2021[214], Lim et al., 2017[273], Ostapiuk and Urbanska, 2022[364]). For that reason, branch-resolving panels and ratios, such as KYNA/QA or 3-HK/KYN ratio, are often more informative than isolated concentrations, even though they too remain imperfect proxies (Fathi et al., 2022[131], Groven et al., 2021[173], Ou et al., 2023[365]).

The KAT branch produces KYNA, a neuromodulatory metabolite whose meaning depends heavily on concentration, compartment, and circuit context. KYNA is often described as protective because it can dampen excitotoxic pressure, yet that description is too narrow (Martos et al., 2022[304], Ostapiuk and Urbanska, 2022[364], Tanaka et al., 2020[471], Tanaka et al., 2025[474]). Excessive KYNA has repeatedly been linked to hypoglutamatergic and cholinergic disruption relevant to cognition, attentional dysfunction, and psychosis-related phenotypes (Erhardt et al., 2017[124], Kozak et al., 2014[242], Martos et al., 2025[303], Potter et al., 2010[392]). It is therefore more accurate to think of KYNA as a tuning molecule than as a universal shield: too little may fail to buffer excitatory stress, whereas too much may suppress signaling in ways that become cognitively or behaviorally costly (Martos et al., 2025[303], Martos et al., 2022[304], Ostapiuk and Urbanska, 2022[364], Szalardy et al., 2012[460], Tanaka et al., 2025[474]). This idea of an “optimal window” fits both experimental and clinical observations. It also explains why opposing interpretations of KYNA can both look plausible if concentration, tissue source, and disease context are not specified (Alves et al., 2024[18], Savitz, 2020[423], Stone et al., 2024[447]). A rise in KYNA under one set of conditions may reflect adaptive buffering, while under another it may signal maladaptive overmodulation (Table 2(Tab. 2); References in Table 2: Caligiore et al., 2022[71]; Cooper and Anders, 1990[90]; Guidetti et al., 2007[174]; Han et al., 2008[182]; Han et al., 2010[181]; Meng et al., 2022[316]; Pinto et al., 2014[386]; Yang et al., 2016[525]) (Choe et al., 2025[86], Knapskog et al., 2023[238], Ostapiuk and Urbanska, 2022[364]). 

The KAT branch is not unitary. A further reason the KYNA-facing branch resists simple interpretation is that “KAT activity” is not a single enzymatic entity (Han et al., 2010[181], Nematollahi et al., 2016[351], Rossi et al., 2019[405]). KAT I-IV differ in tissue distribution, subcellular localization, substrate promiscuity, and likely physiological dominance across compartments, meaning that a measured KYNA signal does not arise from one uniform KAT system (Table 2(Tab. 2)) (Han et al., 2010[181], Wyckelsma et al., 2020[519]). In broad terms, cerebral KYNA is most often discussed through a KAT II-dominant lens, whereas peripheral KYNA may reflect a broader mixture of renal, hepatic, muscular, and mitochondrial aminotransferase biology (Baran et al., 2010[34], Guidetti et al., 2007[174], Juhász et al., 2025[219], Szabó et al., 2025[458]). This matters directly for the KYNA paradox: identical directional changes in KYNA need not imply identical biology if the dominant isoform context differs between blood, CSF, and region-specific brain tissue (Guidetti et al., 1997[175], Rossi et al., 2019[405], Skorobogatov et al., 2021[439]). Framed this way, the apparent contradiction is less a failure of the pathway model than a reminder that KYNA is an enzymatically plural and compartment-conditioned signal (Herédi et al., 2017[191], Rossi et al., 2019[405]). Accordingly, any interpretation of a KYNA-leaning state should specify not only concentration and matrix, but also the most plausible KAT isoform context generating that signal (Amori et al., 2009[19], Skorobogatov et al., 2021[439]). 

The opposing route, shaped by KMO, channels KYN toward intermediates such as 3-HK and 3-HAA and ultimately toward QA, with additional relevance to de novo NAD^+^ synthesis (Amori et al., 2009[19], Castellano-Gonzalez et al., 2019[75], Juhász et al., 2026[218], Phillips et al., 2019[385]). Under inflammatory conditions, this branch is often discussed in relation to oxidative stress, mitochondrial strain, and glutamatergic dysregulation (de la Flor and O'Connor, 2025[104], Pukoli and Vécsei, 2025[395], Zádori et al., 2018[535]). Mechanistic work suggests a time-dependent tradeoff here. Transient KMO engagement may support metabolic adaptation and NAD^+^ requirements, whereas sustained activation appears more likely to favor accumulation of redox-active intermediates, amplification of reactive oxygen species, and reduced mitochondrial reserve (Castellano-Gonzalez et al., 2019[75], de la Flor and O'Connor, 2025[104], Joisten et al., 2021[214]). QA is especially prominent in psychiatric discussions because it is frequently framed as a pro-excitatory metabolite with potential relevance to microglial activation and NMDAR-linked signaling (Hestad et al., 2022[192], Phillips et al., 2019[385], Savitz, 2020[423]). Yet even here, the biology remains context sensitive. Net functional impact depends on compartment, chronicity, surrounding antioxidant capacity, and the balance between KYNA-facing and KMO-facing flux (Amori et al., 2009[19], Ostapiuk and Urbanska, 2022[364], Pukoli and Vécsei, 2025[395]). The real question is therefore not whether one metabolite is intrinsically “good” or “bad,” but what overall branch pattern is being generated under a given inflammatory milieu.

This branch-based framing helps explain why studies using only Trp, KYN, or KYN/Trp ratio often produce apparently divergent interpretations. A shared signal of increased entry into the pathway does not tell us whether the system is moving toward modulatory buffering, toward oxidative and excitatory stress, or toward a mixed and time-dependent adaptation. In psychiatric biomarker research, that distinction matters because downstream metabolites are much closer to the signaling domains that plausibly link immune biology to symptoms. Branch balance is therefore the crucial hinge between upstream inflammatory context and downstream functional consequence.

### 2.3 Downstream effectors relevant to psychiatry

In psychiatry, the importance of the Trp-KYN pathway lies less in metabolite labels themselves than in the functional systems they disrupt or recalibrate (Marx et al., 2021[305], Muneer, 2020[343], Savitz, 2020[423]). Its downstream domains are best understood as consequence spaces rather than direct clinical readouts, because the relevance of branch activity depends on how it reshapes receptor signaling, redox balance, mitochondrial energetics, immune feedback, and symptom-relevant behavior (Cervenka et al., 2017[77], González Esquivel et al., 2017[168], Juhász et al., 2026[218], Pocivavsek et al., 2024[391]). The key question is therefore not simply which metabolite increased, but which biological systems were altered strongly enough to influence cognition, motivation, salience, energy, or stress responsiveness (Marx et al., 2021[305], Skorobogatov et al., 2021[439]). 

One major downstream domain is glutamatergic and cholinergic signaling (Erhardt et al., 2017[124]). The psychiatric importance of the pathway lies less in any simple good-versus-bad metabolite dichotomy than in its capacity to alter NMDAR-relevant tone, α7-nicotinic modulation, and broader circuit-level signal regulation (Erhardt et al., 2017[124], Savitz, 2020[423], Tanaka et al., 2025[472], Wonodi and Schwarcz, 2010[514]). In this framework, Trp-KYN metabolites are best viewed as modulators of synaptic setpoints rather than as direct symptom markers, with likely relevance to cognition, attentional control, salience assignment, and reward-related processing (Erhardt et al., 2017[124], Potter et al., 2010[392], Sapienza et al., 2023[418]). 

A second downstream domain involves oxidative balance and mitochondrial energetics. When inflammatory conditions sustain flux toward redox-active intermediates, experimental systems often show greater reactive oxygen species burden, impaired respiratory capacity, reduced mitochondrial reserve, and heightened vulnerability to excitatory stress (de Lima et al., 2025[105], Figueiredo Godoy et al., 2025[140], Juhász et al., 2025[219], Mor et al., 2021[336], Nagy-Grócz et al., 2024[347], Szabó et al., 2025[459]). This matters clinically because fatigue, psychomotor slowing, low energy, reduced persistence, and cognitive inefficiency are all phenotypes in which inflammatory, metabolic, and motivational processes may converge (Felger and Treadway, 2017[137], Kealy et al., 2020[229], Lacourt et al., 2018[251]). Here again, the pathway is most informative when treated as a systems-level contributor to energetic strain rather than as a collection of isolated metabolite abnormalities. 

A third downstream domain is immune-to-behavior coupling itself. Trp-KYN metabolites should not be viewed only as passive readouts of inflammation, because some also participate in feedback loops that can stabilize or amplify altered immune-metabolic states (Tanaka, 2026[464], Tanaka et al., 2021[475], Tsuji et al., 2023[485], Wirthgen et al., 2017[513]). That makes the pathway especially relevant to transdiagnostic phenotypes such as anhedonia, negative symptoms, stress sensitivity, and inflammation-linked cognitive dysfunction, where immune signaling, circuit modulation, and metabolic strain may interact rather than operate as separate layers (Haroon et al., 2020[184], Hunt et al., 2020[203], Savitz, 2020[423], Tanaka, 2026[464], Tanaka and Battaglia, 2025[469], Tanaka et al., 2025[472]). 

### 2.4 Extended pathway metabolites beyond the canonical kynurenic acid-quinolinic acid framework

Although this review centers on the Trp-KYN metabolites most consistently studied in psychiatric and translational work, the downstream landscape is broader than the canonical KYNA-3-HK-QA axis alone. Several lesser-discussed metabolites deserve brief consideration because they may refine pathway interpretation in a context-, compartment-, and state-dependent manner (Pocivavsek et al., 2024[391], Tanaka et al., 2024[473], Tanaka et al., 2021[475]). Their main value here is not to displace the core branch-balance framework, but to show that the pathway contains additional signaling and metabolic products that may become useful in deeper multi-analyte panels (Cervenka et al., 2017[77], Lim et al., 2017[273], Marx et al., 2021[305]).

Among these, xanthurenic acid (XA) and cinnabarinic acid (CA) are the most promising to mention explicitly (Stone et al., 2013[448]). XA has attracted interest because it extends the KYNA-adjacent side of the pathway and has been linked experimentally to glutamatergic and dopaminergic regulation, including interactions relevant to metabotropic glutamate signaling and frontal cortical function (Maitre et al., 2024[293], Sathyasaikumar et al., 2017[422], Taleb et al., 2021[461]). CA is even more distinctive as a trace KYN with reported mGlu4 agonist activity and possible neuroactive or even antipsychotic-like properties in preclinical work (Fazio et al., 2012[134], Fazio et al., 2014[135], Ulivieri et al., 2020[489]). Neither metabolite has an evidence base comparable to KYNA or QA, but both are useful reminders that KYN-pathway signaling may extend beyond the familiar NMDA-centered contrast and may include additional modulatory products with context-dependent relevance to cognition, salience, and psychosis-related biology (Fazio et al., 2017[133], Stone et al., 2013[448], Tanaka et al., 2021[475]). 

Anthranilic acid (AA) and picolinic acid (PA) are best introduced more cautiously, but they still help widen interpretation beyond a simple protective-versus-toxic binary. AA is a genuine branch metabolite rather than a trivial side product and has emerging signaling relevance, including discussion as a possible receptor-active metabolite in neuropsychiatric contexts (Jiménez-García et al., 2025[211], Oxenkrug, 2024[366], Oxenkrug and Forester, 2024[367]). PA is valuable for a different reason: it complicates the assumption that the downstream KMO-facing arm should be read only through QA and oxidative burden (Aucique-Pérez et al., 2019[24], Beninger et al., 1994[44], Grant et al., 2009[172], Kalisch et al., 1994[223]). In other words, the distal branch is not chemically or functionally monolithic. For this review, both metabolites are best treated as qualifiers that reinforce pathway diversity and as plausible additions to future expanded panels, rather than as current anchors of psychiatric inference.

By contrast, 8-hydroxiquinaldic acid and quinaldic acid (QAA) do not require extended discussion here, but they deserve more than a passing mention. Recent research still places them among the lesser-studied downstream or microbiota-linked KYN derivatives, with intriguing biochemical relevance but far less direct psychiatric evidence than the core metabolites discussed above (Kennedy et al., 2017[231], Shaw et al., 2023[435], Szabó et al., 2025[458]). QAA may nevertheless be of particular interest because, as a downstream product of KYNA, it was reported to be significantly reduced in brain regions of KAT II knockout mice even where KYNA itself did not differ significantly (Szabó et al., 2025[458]). This raises the possibility that QAA could serve as a secondary indicator of KYNA-related pathway activity under some conditions. Conceptually, these metabolites broaden the interpretive landscape of Trp-KYN biology beyond the analytes most commonly measured in psychiatric research and suggest that future gut-aware or high-depth metabolomic studies may uncover additional context-sensitive signals. For now, however, they are best regarded as emerging peripheral or microbial-associated candidates rather than core elements of the minimum translational set.

## 3. Chronic Low-Grade Inflammation as the “Background Field” in Psychiatry

Chronic LGI, often described as metaflammation, can be viewed as the background field in which psychiatric symptoms emerge, shaping immune tone at rest and biasing metabolic pathways such as the Trp-KYN system long before any discrete inflammatory episode is measured (Cervenka et al., 2017[77], Hunt et al., 2020[203]). Operationally, this state may sit within the C-reactive protein (CRP) 3-10 mg/L range yet still keep cortisol- and cytokine-sensitive Trp catabolism partially engaged, making KYN/Trp ratio closer to a set-point marker than a pure event marker. This matters because apparently “baseline” samples are often not biologically neutral; they may already reflect long-standing immunometabolic calibration (Cussotto et al., 2020[97]). To understand why Trp-KYN findings vary across psychiatric studies, it is therefore necessary to identify which real-world exposures most consistently sustain this background inflammatory state in clinical cohorts (Table 3(Tab. 3); References in Table 3: Alme et al., 2021[13]; Almulla et al., 2022[14]; Arroyo-Belmonte et al., 2021[23]; Badawy, 2017[29]; Baysak et al., 2022[40]; Bujtor et al., 2021[65]; Cussotto et al., 2020[97]; Fabbri et al., 2021[126]; Heng et al., 2023[190]; Irwin et al., 2016[206]; Kiank et al., 2010[234]; Kopra et al., 2021[241]; Kozieł and Urbanska, 2023[243]; Kuuskmäe et al., 2023[247]; Leticia Fernandez-Carballo et al., 2021[263]; Lischka et al., 2022[274]; Louvrou et al., 2024[280]; Mallmann et al., 2018[295]; Millischer et al., 2021[328]; Önder et al., 2023[358]; Orhan et al., 2024[360]; Pathak et al., 2020[376]; Paz et al., 2025[380]; Pelletier-Baldelli et al., 2021[382]; Réus et al., 2015[400]; Rykov et al., 2020[408]; Schröder et al., 2021[428]; Sun et al., 2025[457]; Theiler-Schwetz et al., 2023[480]; Zhang et al., 2022[541]; Zhu et al., 2024[546]) (Hunt et al., 2020[203]).

One useful extension of this background-field model is that LGI should not be interpreted independently of concurrent HPA-axis tone. In practice, the psychiatric meaning of a Trp-KYN shift is often clearer when inflammatory markers are read alongside cortisol, because these two upstream signals provide a plausible, if indirect, map of relative IDO- versus TDO-weighted pathway activity (Janssen et al., 2021[210]). A diagnosis-stratified framework based on cortisol-inflammation profiles therefore offers a pragmatic bridge between broad exposure architecture and disorder-level interpretation, while preserving the manuscript's central caution that such profiles are inferential proxies rather than direct measures of enzyme activity or flux (Table 4(Tab. 4); References in Table 4: Beijers et al., 2019[42]; Costello et al., 2019[95]; Fries et al., 2014[151]; Glaus et al., 2018[165]; Hori and Kim, 2019[197]; Jones et al., 2021[215]; Juruena et al., 2018[220]; Kaestner et al., 2005[222]; Lamers et al., 2013[254]; Lyu et al., 2023[286]; Maletic and Raison, 2014[294]; Mazza et al., 2018[307]; Misiak et al., 2021[329]; Mondelli et al., 2010[334]; Mondelli et al., 2015[333]; Nandam et al., 2019[349]; O'Keane et al., 2012[356]; Olff and van Zuiden, 2017[357]; Peruzzolo et al., 2022[384]; Silić et al., 2022[437]; Solmi et al., 2021[443]; Vogelzangs et al., 2013[500]) (Messaoud et al., 2022[318], Muneer, 2020[343], Tanaka et al., 2021[475]).

In psychiatric cohorts, inflammation-linked biomarkers are often interpreted as though they arise mainly from diagnosis-related biology, yet many of the strongest upstream drivers sit outside diagnostic labels (Bhikram and Sandor, 2022[52], Osimo et al., 2018[362], Yuan et al., 2019[534]). Adiposity, smoking, sleep disruption, inactivity, diet, and psychosocial stress can all raise inflammatory tone while also altering Trp availability, KYN production, or downstream branch interpretation (Cussotto et al., 2020[97], Gialluisi et al., 2020[161], Strasser et al., 2015[452], Tanaka and Battaglia, 2025[469]). These influences do not simply add noise. They shift the biological baseline on which later symptom states, medication exposures, and environmental stressors are superimposed (Table 3(Tab. 3)) (Goldsmith et al., 2023[166], Janssen et al., 2021[210], Yuan et al., 2019[534]). LGI should therefore be treated as an explicitly modeled part of study design, stratification, and interpretation rather than as an invisible background nuisance. 

### 3.1 Drivers of low-grade inflammation that matter in psychiatric cohorts 

Among the strongest and most pervasive drivers of LGI in psychiatric cohorts is adiposity with associated metabolic dysregulation, particularly in chronic depression and schizophrenia spectrum disorders (Carli et al., 2021[74], Oracz et al., 2025[359], Zwiep et al., 2025[548]). Visceral adiposity behaves like an immune organ: macrophage skewing and adipokine imbalance raise CRP, interleukin 6 (IL-6), and tumor necrosis factor-alpha (TNF-α) while shifting Trp handling toward higher KYN and KYN/Trp ratio (Huet et al., 2021[202], Kang et al., 2016[225], Ren et al., 2022[399]). In some studies, these profiles also align with more severe or TRD presentations (Lv et al., 2025[285], Molina et al., 2021[331], Zwiep et al., 2025[548]). The key point is not simply that obesity and psychiatric illness co-occur, but that metabolic burden changes the biological starting conditions under which inflammatory and KYN-related findings are observed (Cussotto et al., 2020[97], Lamers et al., 2018[252], Tanaka et al., 2021[475]). In psychosis, this problem is often amplified by treatment exposure. Antipsychotic-associated weight gain can further increase cytokine tone and cardiometabolic burden, making it difficult to separate illness-related inflammatory signals from treatment-shaped physiology if metabolic status is not modeled directly (Lamers et al., 2018[252], Naudé et al., 2025[350], Zwiep et al., 2025[548]). The body mass index (BMI) alone is therefore often too crude; waist measures, recent weight change, and broader metabolic phenotyping are more informative because central adiposity and insulin resistance can each alter pathway readouts (Cussotto et al., 2020[97], Huet et al., 2021[202], Zwiep et al., 2025[548]). Metabolic variables should travel with immune and KYN measures from the outset rather than being added after interpretation has already drifted.

Smoking and persistent sleep disruption form another high-impact exposure cluster. Both are common in TRD and psychosis populations, both can amplify inflammatory signaling, and both can distort the apparent relationship between biomarkers and symptom severity (Galan et al., 2022[155], Kindler et al., 2020[237], Mancuso et al., 2023[296]). Smoking is not background noise in blood-based Trp-KYN studies; it is a necessary covariate, especially when comparison groups differ in prevalence or intensity (Bose et al., 2026[56], Galan et al., 2022[155], Kindler et al., 2020[237]). Sleep contributes a partly independent layer: altered sleep duration, insomnia, hypersomnia, and circadian disruption are linked to higher CRP and IL-6 even after accounting for BMI and smoking, suggesting that disturbed sleep can carry its own inflammatory imprint (Kuwano et al., 2018[248], Yilmaz et al., 2022[532]). This matters because sleep is often treated only as a symptom of illness while simultaneously acting as a driver of biomarker variance (Kuwano et al., 2018[248], Sapienza et al., 2025[417], Yilmaz et al., 2022[532]). When smoking and sleep disruption cluster together, as they often do in real-world cohorts, inflammation can appear more diagnosis-specific than it truly is (Mancuso et al., 2023[296], Milaneschi et al., 2021[322], Milaneschi et al., 2021[323]). Some apparently inconsistent cytokines or KYN findings become more coherent once these moderators are modeled directly (Galan et al., 2022[155], Medic et al., 2017[311], Milaneschi et al., 2021[322]).

Physical inactivity and diet add further variance to the same background field. Sedentary behavior contributes to metaflammation, whereas exercise can alter peripheral KYN handling and bias conversion toward KYNA in muscle, making it important to distinguish chronic inactivity from recent exertion around sampling (Cervenka et al., 2017[77], Tanaka et al., 2021[475], Westbrook et al., 2026[507]). Diet works more slowly but no less meaningfully. Western or ultra-processed dietary patterns can heighten immune tone, whereas Mediterranean-style patterns tend to track lower CRP and IL-6 (Chehadi et al., 2026[81], Cobos-Palacios et al., 2022[89], Francis et al., 2022[146], Rangel et al., 2025[397]). Diet also influences Trp availability more broadly, so fasting status alone does not resolve longer-term dietary confounding when baseline pathway interpretation is the goal (Chehadi et al., 2026[81], Lim et al., 2021[272], Liu et al., 2019[276], Rangel et al., 2025[397]). These exposures are easy to undermeasure because they are often recorded superficially or omitted altogether, yet they can substantially reshape apparent baseline Trp-KYN biology across cohorts (Dugué et al., 2023[113], Kiluk et al., 2021[235], Lim et al., 2021[272]). In effect, inactivity and diet regulate the thermostat on which more visible inflammatory or psychiatric processes are later layered (Cervenka et al., 2017[77], Sun et al., 2023[456], Tanaka et al., 2021[475]).

Psychosocial stress is another major component of this background field because chronic stress can sustain LGI while also pushing Trp catabolism from two directions: through stress-linked TDO programs and through stress-evoked cytokine/IDO signaling (de Bartolomeis et al., 2025[101], Fuertig et al., 2016[152], Michels et al., 2018[321]). This dual-route architecture makes stress especially relevant in psychiatric cohorts marked by adversity exposure, ongoing social threat, hospitalization, or repeated stress sensitization (Goldsmith et al., 2023[166]). It also helps explain why symptom modules such as anhedonia, fatigue, threat reactivity, and cognition may map onto inflammatory and KYN signals more clearly than diagnosis labels alone (Fourrier et al., 2019[145], Goldsmith et al., 2023[166], Lucido et al., 2021[283], Tanaka and Battaglia, 2025[469]). It often coexists with smoking, sleep disruption, metabolic strain, and medication exposure, deepening the problem of stacked confounding (Goldsmith et al., 2023[166], Haroon et al., 2020[184], Vancassel et al., 2018[497]). A more realistic view is that chronic LGI in psychiatry often reflects interacting exposure constellations rather than single dominant causes (Goldsmith et al., 2023[166], Haroon et al., 2020[184]). That is precisely why nominally similar diagnostic groups can produce sharply different biomarker profiles across studies.

### 3.2 Why symptom modules often beat diagnoses 

Because metaflammation is shaped by exposures that cut across diagnostic boundaries, immune-metabolic biology often aligns more tightly with symptom modules than with categorical diagnoses, particularly in disorders as heterogeneous as TRD and schizophrenia spectrum conditions (de Kluiver et al., 2023[103], McQuaid, 2021[310], Penninx et al., 2025[383]). Longitudinal work suggests that atypical, energy-balance-leaning depressive profiles track CRP, IL-6, adiposity, and metabolomic shifts more reliably than the broad MDD label, while polygenic risk for CRP or BMI maps more strongly onto appetite and fatigue nodes than onto the full syndrome (de Kluiver et al., 2023[103], Kappelmann et al., 2021[226], Lamers et al., 2020[253]). Across severe mental illness, multi-omics signatures often distinguish patients from controls yet fail to cleanly separate diagnoses, whereas immune differences more often track dimensional severity, cognition, and motivation (Hagenberg et al., 2025[179], Naifar et al., 2025[348], Solomon et al., 2025[444], Tanaka, 2025[462], Tanaka, 2025[463]). The most reproducible modules in this space resemble sickness behavior and energy-reward disruption, dimensions that can be quantified across disorders and mapped onto Trp-KYN flux hypotheses (Brydges et al., 2022[63], de Kluiver et al., 2023[103], Penninx et al., 2025[383]).

Four symptom modules repeatedly emerge as the strongest candidates for immune-metabolic coupling: anhedonia or reward dysfunction, fatigue with sickness-behavior-like symptoms, cognitive slowing with executive dysfunction, and, more cautiously, suicidality as a cross-diagnostic outcome (Brydges et al., 2022[63], Lamers et al., 2018[252], Penninx et al., 2025[383]). Among these, anhedonia and fatigue are the most consistently informative (de Kluiver et al., 2023[103], Milaneschi et al., 2021[323], Penninx et al., 2025[383]). The immune-metabolic signal appears to land especially hard on reward motivation and behavioral effort: higher IL-6 and CRP, immune-cell insulin resistance, and altered amino-acid and energetic handling align with reduced reward pursuit and ventral striatal dysfunction (Bekhbat et al., 2025[43], Felger et al., 2016[136], Lucido et al., 2021[283]). Fatigue is the archetypal metaflammation readout, with sleepiness, hypersomnia, hyperphagia, leaden paralysis, and low drive tracking inflammatory and cardiometabolic indices over time (de Kluiver et al., 2023[103], Milaneschi et al., 2020[324], Penninx et al., 2025[383]). In practice, fatigue and anhedonia often co-occur as a single high-yield module, flagging patients most likely to carry combined immune and metabolic dysregulation and, plausibly, shifted Trp partitioning toward KYNs (Haroon et al., 2020[184], Tanaka et al., 2021[475], Zwiep et al., 2026[547]). 

Cognition is a more equivocal endpoint in blood-based studies, with meta-analytic synthesis suggesting only weak associations between peripheral immune markers, including KYNs, and cognitive domains, implying measurement noise, compartment mismatch, or subgroup effects (Morrens et al., 2022[340], Sapienza et al., 2024[415], Török et al., 2020[483]). Suicidality should be handled with similar caution: some genetic and biomarker work links IL-6 signaling to suicide risk, and anhedonic states are clinically associated with higher risk, yet suicidality remains multiply determined and far from inflammation-specific (Almulla et al., 2022[16], Bora, 2019[55], Brydges et al., 2022[63]). Even so, the broader lesson holds: symptom modules usually provide a more biologically coherent target than diagnoses when the goal is to link LGI and Trp-KYN activity to clinical presentation (Patlola et al., 2023[378], Sapienza et al., 2025[417], Strasser et al., 2017[451]). The next question is therefore methodological: do current biomarker strategies capture pathway activity with enough resolution to connect immune context to these phenotypes?

## 4. Measurement and Interpretation: What Tryptophan–Kynurenine Biomarkers Can Actually Tell Us

Trp-KYN biomarkers are often treated as straightforward readouts of “inflammation-to-brain chemistry,” yet in practice they provide context-dependent snapshots of a distributed pathway whose meaning depends on panel depth, sampling matrix, timing, and confounding structure (Coppens et al., 2022[92], Skorobogatov et al., 2021[439], Tanaka et al., 2021[475]). Across mood and psychosis literatures, even robust signals such as lower Trp or higher KYN/Trp ratio can reflect transport biology, albumin binding, renal handling, stress-endocrine effects, or inflammatory catabolism in different mixtures across cohorts (Coppens et al., 2022[92], Karu et al., 2016[227], Skorobogatov et al., 2021[439]). Because only a subset of analytes reliably map across compartments, peripheral profiles cannot be naively equated with central branch (Almulla et al., 2022[14], Inam et al., 2023[205], Skorobogatov et al., 2021[439]). The practical goal of this section is therefore not to nominate a single best biomarker, but to define interpretive rules that treat Trp-KYN panels as network fingerprints rather than verdicts (Coppens et al., 2022[92], Haroon et al., 2020[184], Skorobogatov et al., 2021[439]). That framing matters because many apparent contradictions in the literature are not true biological oppositions; they are mismatches between what was measured, where it was measured, when it was measured, and what background exposures were allowed to shape the signal before interpretation began (Arnone et al., 2018[22], Marx et al., 2021[305], Skorobogatov et al., 2021[439]).

### 4.1 Core markers vs undermeasured markers

Most psychiatric studies quantify Trp, KYN, and the KYN/Trp ratio, a pragmatic core trio that primarily captures entry-level diversion of Trp into KYN production rather than downstream pathway consequences (Badawy and Guillemin, 2019[30], Marx et al., 2021[305], Tanaka and Vécsei, 2021[477]). Trp reflects substrate availability and is sensitive to protein binding, transport, diet, systemic physiological state, and acute nutritional timing. Absolute KYN varies more across phenotypes and matrices, but it too remains a broad readout. KYN/Trp ratio is best read as a coarse index of catabolic engagement that integrates immune-inducible IDO programs, hepatic TDO influences, and whole-body Trp flux; it is not a clean proxy for one enzyme and should not be labeled as such (Badawy, 2017[27], Badawy and Guillemin, 2019[30]). Used carefully, this core panel can indicate whether the pathway gate appears more open or more closed, especially when interpreted alongside inflammatory markers, medication exposure, metabolic status, and renal function. Its obvious limitation is that entry-level diversion does not reveal whether KYN is being routed toward modulatory products, redox-active intermediates, or NAD^+^-linked endpoints (Badawy, 2017[27]). In other words, the core trio is useful for detecting pathway entry, but it is biologically thin if the study question concerns neuroactive or branch-specific consequences.

That limitation is precisely why downstream metabolites such as KYNA, 3-HK, and QA deserve greater emphasis (Fujigaki et al., 2017[154], Leclercq et al., 2021[261], Pires et al., 2022[388]). These analytes add branch-resolving information because they sit closer to receptor-level, redox, and immune-feedback consequences than Trp or KYN alone (Fujigaki et al., 2017[154], Meier and Savitz, 2022[314], Pires et al., 2022[388]). KYNA is informative for modulatory and antagonistic signaling contexts, especially where glutamatergic and α7-nicotinic mechanisms are implicated (Erhardt et al., 2009[122], Erhardt et al., 2017[124], Fujigaki et al., 2017[154]). By contrast, 3-HK and QA more often anchor interpretations related to oxidative load, inflammatory routing, mitochondrial strain, and NMDAR-relevant biology (Fujigaki et al., 2017[154], Pires et al., 2022[388], Wurfel et al., 2017[518]). Psychiatric studies that stop at Trp, KYN, and KYN/Trp ratio therefore capture pathway entry but not the downstream pattern most likely to matter biologically (de Bartolomeis et al., 2025[101], Hunt et al., 2020[203], Marx et al., 2021[305]). A branch-resolving panel does not need to be exhaustive to be useful, but it should move beyond the core trio if the aim is mechanistic interpretation rather than descriptive profiling (de Bartolomeis et al., 2025[101], Inam et al., 2023[205], Marx et al., 2021[305]). This becomes especially important when the same KYN/Trp ratio signal could coexist with either a KYNA-leaning or QA-leaning pattern, producing very different biological implications despite a similar entry-level readout (Inam et al., 2023[205], Marx et al., 2021[305], Wurfel et al., 2017[518]).

A practical way to think about panel depth is tiered measurement. Tier 1 panels quantify Trp, KYN, and KYN/Trp ratio and are useful for screening catabolic engagement (Haroon et al., 2020[184], Hunt et al., 2020[203], Yan et al., 2023[523]). Tier 2 panels add branch-resolving metabolites such as KYNA, 3-HK, and QA, allowing more directional interpretation (Haroon et al., 2020[184], Skorobogatov et al., 2021[439], Yan et al., 2023[523]). Tier 3 designs extend further by pairing these metabolites with inflammatory phenotyping, matched matrices, or longitudinal sampling (Haroon et al., 2020[184], Skorobogatov et al., 2021[439], Yan et al., 2023[523]). In psychiatric biomarker work. Tier 2 should be viewed as the minimum target when branch interpretation is central to the study question (Table 5(Tab. 5); Haroon et al., 2020[184], Inam et al., 2023[205], Ou et al., 2023[365]; References in Table 5: Abujrais et al., 2025[3]; Al Saedi et al., 2022[8]; Brandi et al., 2022[57]; Chawdhury et al., 2021[80]; Chen et al., 2021[82]; de Jong et al., 2009[102]; Eggertsen et al., 2023[118]; Fathi et al., 2022[130]; Fuertig et al., 2016[153]; Holthuijsen et al., 2024[196]; Juhász et al., 2026[218]; Meier et al., 2016[313]; Metri et al., 2023[319]; Nadour et al., 2022[345]; Rodrigues et al., 2021[404]; Saliba et al., 2025[412]; Skorobogatov et al., 2021[439]; Ye et al., 2025[530]). The value of this framework is not that it imposes a rigid standard on every study design, but that it forces a match between biological ambition and analytic depth. If a study wants to argue that inflammation shifts branch routing, it must measure branch routing rather than infer it from the gate alone.

Even when KYNA is measured, interpretation remains incomplete if the analyte is treated as isoform-neutral (Han et al., 2010[181], Nematollahi et al., 2016[351], Rossi et al., 2019[405]). KYNA compresses the output of multiple KAT systems whose biological meaning is unlikely to be equivalent across compartments (Han et al., 2010[181], Rossi et al., 2019[405], Yu et al., 2006[533]). A peripheral KYNA value may be shaped more by renal, hepatic, muscular, or broader aminotransferase biology, whereas a CSF or region-specific brain value is more plausibly linked to cerebral KAT-dominant regulation (Baran et al., 2010[34], Han et al., 2010[181], Nematollahi et al., 2016[351]). For that reason, KYNA should be interpreted with an explicit compartment-and-isoform frame rather than as a portable surrogate of the same process across blood and brain (Orhan et al., 2024[360], Pocivavsek et al., 2024[391], Skorobogatov et al., 2021[439]). 

### 4.2 Ratios and “flux thinking” (with guardrails)

Ratios are attractive because they compress pathway relationships into tractable summary measures, but they remain proxies rather than direct readouts of enzyme activity or true metabolic flux (de Mas and Cascante, 2019[106], Law et al., 2022[259], Park et al., 2016[372]). Their value lies in showing how analytes move relative to one another under a defined sampling context (Di Filippo et al., 2022[110], Law et al., 2022[259], Park et al., 2016[372]). Their weakness is that the same ratio may rise or fall for more than one biological reason, including changes in substrate availability, clearance, transport, or branch competition (Law et al., 2022[259], Noor et al., 2016[353], Park et al., 2016[372]). Ratios are therefore most useful when interpreted alongside absolute concentrations, matrix, timing, and co-measured immune indices rather than as stand-alone verdicts (Di Minno et al., 2022[111], Park et al., 2016[372]). A ratio can summarize a pathway relationship, but it cannot replace knowledge of compartment, physiology, or pre-analytics (Di Minno et al., 2022[111], Li et al., 2024[267], Park et al., 2016[372]). This is one of the field's recurring interpretive errors: compact summary measures are often treated as though they directly assay hidden enzymology, when in reality they support only constrained inference (Hackett et al., 2016[178], Law et al., 2022[259], Shin et al., 2026[436]). 

**Figure 3:**
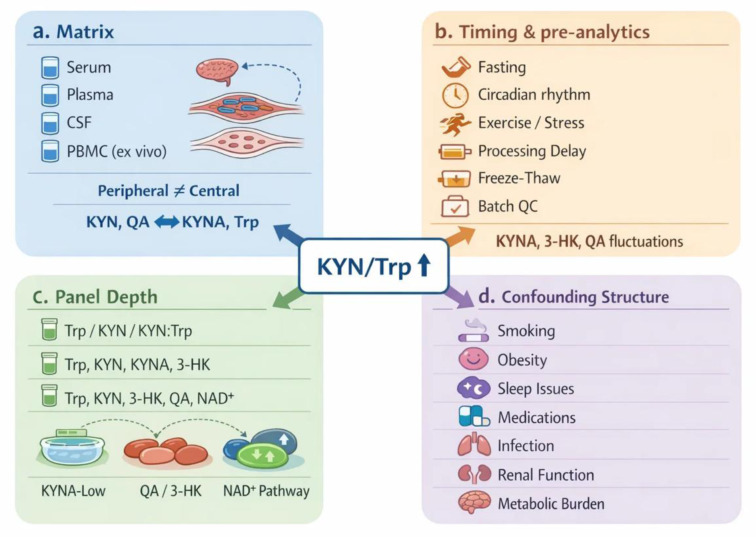
Why studies disagree: how matrix, timing, panel depth, and confounding reshape interpretation of elevated KYN/Trp ratios. Schematic summary showing that identical increases in KYN/Trp ratio can carry different biological meanings depending on the interpretive framework. Differences in biospecimen matrix, sampling timing and pre-analytics, downstream metabolite coverage, and cohort-level confounding can shift inference toward peripheral catabolic activation, central branch imbalance, redox-inflammatory burden, or altered NAD^+^-related flux. Thus, apparent disagreement across studies may reflect variation in measurement context and analytical depth rather than genuinely contradictory Trp-KYN biology. 3-HK, 3-hydroxykynurenine; CSF, cerebrospinal fluid; KYN, kynurenine; KYN/Trp, kynurenine-to-tryptophan ratio; KYNA, kynurenic acid; NAD^+^, nicotinamide adenine dinucleotide; PBMC, peripheral blood mononuclear cell; QA, quinolinic acid; Trp, tryptophan.

KYN/Trp ratio illustrates both the utility and the common misinterpretation of ratio thinking (Badawy and Guillemin, 2019[30], Jamshed et al., 2022[208], Strasser et al., 2017[451]). It is a reasonable proxy for increased Trp catabolism into KYN, but it cannot determine which gatekeeper dominates, whether the shift is primarily immune- or stress-linked, or which downstream branch consequences are emerging (Badawy, 2017[27], Badawy and Guillemin, 2019[30], Yan et al., 2024[522]). The same ratio may rise because cytokines induce extrahepatic IDO, because glucocorticoid tone alters TDO-related flux, or because free Trp falls while KYN is buffered by slower clearance and downstream bottlenecks (Badawy and Guillemin, 2019[30], Hunt et al., 2020[203], Jamshed et al., 2022[208]). To move from “catabolism increased” to “branch biology changed,” investigators usually need branch-balance ratios such as KYNA/QA ratio and practical KMO-tilt indicators (Almulla et al., 2022[15], Almulla et al., 2022[16], Tanaka et al., 2021[475]). Even these require guardrails. Branch competition means rerouting can alter ratios without implying more enzyme, and peripheral ratios may not mirror central balance. Partial panels are therefore a major source of apparent contradiction, because similar KYN/Trp ratio signals can coexist with opposite downstream patterns (Figure 3(Fig. 3)) (Almulla et al., 2022[14], Almulla et al., 2022[15], Almulla et al., 2022[16]). The practical lesson is straightforward: ratio thinking becomes stronger as panel depth improves, but misleading when shallow panels are asked to answer mechanistic questions they were never designed to resolve (Badawy, 2017[27], Badawy and Guillemin, 2019[30], Strasser et al., 2017[451]).

Biospecimen choice imposes both biological and analytical constraints. Serum and plasma can differ systematically, CSF is rarer but is often interpreted as closer to central processes, and PBMC or ex vivo paradigms probe cellular capacity rather than steady-state circulating levels (Eggertsen et al., 2023[118], Heng et al., 2023[190], Skorobogatov et al., 2021[439]). Apparent matrix effects can reflect genuine compartment biology, but they can also arise from pre-analytics and platform variation (Eggertsen et al., 2023[118], Heng et al., 2023[190], Sens et al., 2023[430]). The safest interpretive rule is therefore simple: matrix should be treated as part of the biological question, not as an interchangeable container (González-Domínguez et al., 2020[169], Heng et al., 2023[190], Metri et al., 2023[319], Tanaka et al., 2025[470]). Peripheral blood may track KYN and sometimes 3-HK reasonably well, but it is less reliable for inferring central KYNA-facing versus QA-facing balance (Jacobs et al., 2019[207], Rodrigues et al., 2021[404], Skorobogatov et al., 2021[439]). CSF helps, but it is not a magic mirror. KYN and QA may show some blood-CSF concordance, whereas KYNA and Trp can be strikingly discordant, so plasma cannot be assumed to proxy brain branch balance (Jacobs et al., 2019[207], Rodrigues et al., 2021[404], Skorobogatov et al., 2021[439]). One underappreciated reason for this dissociation is that KYNA in different matrices may reflect different dominant KAT isoform environments rather than a single shared branch state sampled at varying distance from the brain (Garrison et al., 2018[158], Nagao et al., 2026[346], Skorobogatov et al., 2021[439]). In that sense, the central-peripheral gap is not merely a transport problem, but also an enzyme-context problem (Garrison et al., 2018[158], Skorobogatov et al., 2021[439], Stone and Williams, 2024[450]). PBMC measures add complementary information, yet they index immune-cell programming more than whole-body in vivo flux (Eminel et al., 2017[120], Jones et al., 2015[216], Skorobogatov et al., 2021[439]). Their value is real, but different: they are better interpreted as markers of inducibility or pathway capacity than as direct surrogates of circulating metabolite levels (Jones et al., 2015[216], Krupa and Kowalska, 2021[244], Skorobogatov et al., 2021[439]).

Three recurring misreads follow from matrix choice. First, serum-plasma differences and delayed processing can mimic diagnosis effects by selectively perturbing unstable intermediates (Hagn et al., 2024[180], Heng et al., 2023[190], Liu et al., 2018[278]). Second, peripheral measures can legitimately track KYN availability while failing to track central branch balance (Jacobs et al., 2019[207], Paul et al., 2022[379], Skorobogatov et al., 2021[439]). Third, PBMC and ex vivo outputs can be mistaken for circulating steady-state biology when they reflect immune programming under stimulated or assay-specific conditions (Bremer et al., 2023[59], Heng et al., 2023[190], Wilson et al., 2025[512]). None of these problems makes a given matrix unusable; the point is that matrix choice changes the meaning of the data (Hagn et al., 2024[180], Heng et al., 2023[190], Liu et al., 2018[278]). Studies are most interpretable when the compartment is built into the hypothesis from the start and then carried transparently through the title, methods, ratios, and claims (Bremer et al., 2023[59], Liu et al., 2018[278], Skorobogatov et al., 2021[439]). 

Timing and pre-analytics can be just as decisive as matrix. Trp-KYN measures are sensitive to fasting status, diurnal phase, recent sleep disruption, acute stress, exercise, processing delay, storage conditions, freeze-thaw exposure, and batch handling (Juhas et al., 2024[217], La Torre et al., 2021[250], Louvrou et al., 2024[280]). Even short fasting windows can alter Trp and selectively shift downstream metabolites, making “baseline” partly a nutritional and circadian timestamp rather than a stable trait (Juhas et al., 2024[217], Louvrou et al., 2024[280], Solvang et al., 2022[445]). Add acute physiology, and the profile becomes even more labile: endurance exercise, resistance training, interval work, and thermal stress can all shift KYNA, QA, and relevant ratios within hours (Joisten et al., 2020[213], Juhas et al., 2024[217], Louvrou et al., 2025[279]). Redox-active intermediates such as 3-HK and 3-HAA are especially vulnerable to drift when sample separation is delayed or temperature handling is suboptimal (Heng et al., 2023[190], Hustad et al., 2012[204], Schwieler et al., 2020[429]). Weak reporting of storage, freeze-thaw cycles, or batch QC can therefore manufacture case-control differences that look biological (Hustad et al., 2012[204]). For this reason, fasting, clock time, recent exertion or stress exposure, and QC procedures should be treated as design variables rather than optional metadata (Anton et al., 2015[21], Juhas et al., 2024[217], Louvrou et al., 2024[280]). The difference between a credible biomarker study and a noisy one is often not the assay platform, but whether these timing-dependent distortions were anticipated rather than discovered too late (Heng et al., 2023[190], Liang et al., 2025[270], Wu et al., 2024[517]). 

### 4.3 Confounders that can dominate the signal

Some confounders are so influential that they should be treated as a universal minimum adjustment set in psychiatric Trp-KYN studies (Coppens et al., 2022[92], Marx et al., 2021[305], Milaneschi et al., 2021[323]). At minimum, analyses should account for adiposity or metabolic status, smoking, medication exposure, renal function, recent infection or inflammatory illness, and sampling state, with sleep disturbance and recent exertion added whenever feasible (Coppens et al., 2022[92], Farup et al., 2023[129], Fellendorf et al., 2021[139]). These variables do not merely polish the model; they can dominate the signal and change what the same biomarker pattern appears to mean (Table 3(Tab. 3)) (Farup et al., 2023[129], Fellendorf et al., 2021[139], Vidal et al., 2020[499]). Inflammation-linked psychiatric studies often fail not because the assays are poor, but because the biological context was undermeasured (Coppens et al., 2022[92], Marx et al., 2021[305], Tanaka et al., 2021[475]). Table 5(Tab. 5) is designed to prevent exactly that problem. It operationalizes the interpretive rules above by specifying the minimal branch-resolving panel and the minimal metadata needed to make Trp-KYN findings comparable and mechanistically interpretable across cohorts (Table 5(Tab. 5)).

Drugs and biology also edit the pathway directly: immune-activating therapies and infections can swamp psychiatric effects by producing large Trp drops and KYN rises, while common agents may modulate TDO- or IDO-related routing more subtly (Larkin et al., 2016[256], Tanaka et al., 2021[475], Wang et al., 2015[505]). Hormonal milieu matters as well, since reproductive state and sex-hormone context can influence Trp routing and immune sensitivity (Badawy, 2017[27], Dunn et al., 2024[117], Hoffmann et al., 2023[194]). None of these variables should be treated as peripheral background detail when the pathway itself is the object of inference (Chen and Guillemin, 2009[84], Tanaka et al., 2021[475], Wang et al., 2015[505]). If the study question centers on psychiatric biology, then competing biological explanations for the same signal have to be made visible, not left implicit (Salminen, 2022[413], Stone and Williams, 2024[450], Tanaka et al., 2021[475]). 

Confounding structure also differs systematically by cohort. In TRD, adiposity, atypical energy symptoms, sleep disturbance, and antidepressant exposure often cluster together (McIntyre et al., 2023[309], Penninx et al., 2025[383], Vreijling et al., 2024[502]). In first-episode psychosis, smoking, acute stress, and recent treatment initiation may dominate (Parksepp et al., 2022[373], Śmierciak et al., 2021[440], Smith et al., 2020[441]). In chronic SCZ, long-term antipsychotic exposure, metabolic burden, and sedentary behavior become especially important (Burschinski et al., 2023[66], Correll et al., 2018[93], Wang et al., 2024[504]). These recurring constellations matter because they can manufacture between-study disagreement even when the underlying pathway biology is not truly contradictory (Schmaal, 2023[426], Schnack and Kahn, 2016[427], Yao et al., 2025[528]). The task is therefore not to eliminate all confounding, which is impossible, but to prespecify the dominant distortion profile for each cohort and model it transparently (Marquand et al., 2019[299], Marquand et al., 2016[300], Schmaal, 2023[426]). A psychiatric biomarker study that ignores cohort-shaped distortion profiles is not merely incomplete; it risks overreading diagnosis-linked effects that are actually being driven by the exposure architecture described in Section 3.

In practice, apparent inconsistencies across psychiatric Trp-KYN studies often reduce to four interpretive axes: matrix, timing, panel depth, and confounding structure. The same entry-level signal can therefore imply different biology depending on where it was measured, when it was sampled, how deeply the pathway was profiled, and which cohort-level distortions were modeled. Figure 3(Fig. 3) summarizes this logic visually, while Table 5(Tab. 5) translates it into a practical design framework. With these guardrails in place, the next step is to ask what the clinical literature actually shows once biomarkers are interpreted in context rather than in isolation.

## 5. Clinical Synthesis across Psychiatric Disorders and Symptom Modules

Clinical studies linking immune activation, chronic low-grade inflammation, and Trp to KYN pathway dynamics span multiple diagnoses, but they become easier to interpret when the evidence is organized using a stable template: immune context, pathway signature, symptom module links, treatment implications, and disorder-specific gaps (Hunt et al., 2020[203], Strasser et al., 2017[451], Tanaka et al., 2021[475]). That structure matters because the pathway is a distributed network, and its metabolites behave like a language with dialects (Badawy, 2017[27], Cervenka et al., 2017[77], Stone and Williams, 2024[450]). The same upward shift in KYN relative to Trp can reflect cytokine-driven induction of IDOs, glucocorticoid-biased hepatic catabolism through TDO, altered peripheral clearance, microbiome-linked rerouting, or a change in albumin binding and substrate availability, depending on what else was measured and in which compartment (Badawy, 2017[27], Badawy and Guillemin, 2019[30], Riazati et al., 2022[402]). In this section, recurring patterns therefore refer to constellations that show up across studies, not to fingerprints tied to a single diagnosis, and they are read through guardrails that keep mechanism separate from proxy (Almulla et al., 2022[15], Badawy and Guillemin, 2019[30], Sales et al., 2023[411]). 

A second lens is module-aware. Reward and motivation disruption, low energy and fatigue, cognitive slowing, and stress-linked sickness features often map onto immune-to-KYN coupling more tightly than labels like MDD or SCZ (Dantzer, 2017[99], Haroon et al., 2020[184], Ormstad et al., 2020[361]). That is why quasi experimental immune activation models, including IFN-based exposures in medical contexts, serve as reference signatures for what robust pathway engagement looks like in humans (Hunt et al., 2020[203], Raison et al., 2010[396], Sforzini et al., 2019[434]). When that reference pattern is kept in mind, the clinical literature across psychiatry stops looking contradictory and starts looking like a set of partial views, each shaped by sampling time, metabolic status, treatment exposure, and the depth of the metabolite panel (Figure 3(Fig. 3)).

### 5.1 Minimal comparability checklist for interpreting clinical tryptophan-kynurenine signals 

Before moving into disorder-specific narratives, it helps to adopt a minimal comparability frame. Peripheral blood matrices are not interchangeable. Plasma, serum, and whole blood differ in handling, clotting effects, and platelet contributions, and these technical choices can shift absolute levels and ratios (Heng et al., 2023[190], Metri et al., 2023[319], Saccaro et al., 2021[409]). Central readouts are even more sensitive. CSF measures, when available, provide a more defensible handle on brain-relevant KYNA and QA, but CSF is scarce and often limited to a narrow set of analytes (Inam et al., 2023[205], Marx et al., 2021[305], Yan et al., 2023[523]). Timing is equally decisive. Sampling in the morning after fasting, compared with afternoon sampling after meals or after a night of poor sleep, can yield different Trp availability and different apparent pathway flux (Brum et al., 2023[62], Hunt et al., 2020[203], Juhas et al., 2024[217]). Acute infections, vaccinations, intense exercise, and psychosocial stress around hospitalization can transiently amplify immune tone and shift KYN dynamics, which can then be misread as trait biology (Milaneschi et al., 2021[323] , Paul et al., 2022[379], Salagre et al., 2023[410]). Minimum branch-resolving Trp-KYN panel (psychiatric cohorts): Trp, KYN, KYNA, QA, and 3-HK, reported with KYN/Trp, KYNA/QA, and 3-HK/KYN ratios, plus CRP and at least one IFN-proximal immune marker when feasible (Table 5(Tab. 5)).

Medication context is another determinant. Antidepressants, antipsychotics, and mood stabilizers reshape weight, glucose and lipid biology, and inflammatory set points, and they may also influence enzymes and transporters that regulate Trp and KYNs (Pu et al., 2022[394], Ruiz-Sastre et al., 2024[407], Sepúlveda-Lizcano et al., 2023[431]). Anti-inflammatory agents, steroids, and immunotherapies more directly perturb upstream drivers and can decouple symptoms from biomarkers, producing clinical improvement without full pathway normalization or pathway changes without immediate symptom relief (Dellink et al., 2025[108], Du et al., 2024[112], Ruiz-Sastre et al., 2024[407]). Inflammatory stratification often decides whether results are interpretable. Baseline hsCRP, IL-6, tumor necrosis factor (TNF), or a composite immune index should be treated as core context, not an optional (Mancuso et al., 2023[296], Pinzi et al., 2025[387], Yang et al., 2019[524]). Without it, a strong effect confined to an immune-high subgroup can disappear into the average (Dellink et al., 2025[108], Reininghaus et al., 2018[398], Yang et al., 2019[524]). 

Ratio discipline is also important. KYN to Trp is widely used as an index of pathway entry and is sometimes described as an IDO proxy (Almulla et al., 2022[16], Badawy and Guillemin, 2019[30], Bartoli et al., 2022[35]). It is safer to interpret it as a flux indicator that is sensitive to multiple influences, including TDO activity, cortisol, diet, hepatic function, and albumin binding (Badawy, 2017[29], Badawy and Guillemin, 2019[30], Messaoud et al., 2019[317]). Downstream balance is often closer to the clinical question, but it is harder to measure well (Marx et al., 2021[305]). KYNA to QA, KYNA relative to 3-HK, or QA relative to PA can hint at branch preference, yet peripheral signals do not always mirror central routing (Bartoli et al., 2022[35], Paul et al., 2022[379], Skorobogatov et al., 2021[439]). Outcomes also need to align with modules. Total symptom scores blend biology-linked features with symptoms that are mechanistically heterogeneous (Bartoli et al., 2022[35], Hunt et al., 2020[203], Marx et al., 2021[305]). When the question is whether immune-driven KYN shifts track fatigue, anhedonia, cognitive efficiency, or suicidal risk, module-anchored outcomes usually outperform total scores (Bartoli et al., 2022[35], Haroon et al., 2020[184], Hunt et al., 2020[203]).

### 5.2 Major depressive disorder and treatment-resistant depression: module map 

In major depressive disorder, immune activation is unevenly. Higher inflammatory burden clusters with metabolic dysregulation, sleep disturbance, pain, and illness chronicity, features that are frequently overrepresented in treatment-resistant presentations (Mancuso et al., 2023[296], Penninx et al., 2025[383], Zwiep et al., 2025[548]). That distribution can make group-level signals for cytokines and acute-phase proteins look like subgroup effects in some datasets, yet like a population shift in others, depending on fasting status, adjustment for adiposity and smoking, capture of insulin resistance, and separation of medication-free from medicated episodes (Beurel et al., 2020[50], Mac Giollabhui et al., 2021[287], Musinguzi et al., 2018[344]). Against this immune backdrop, the most consistent pathway entry finding is Trp depletion (Almulla et al., 2022[14], Mancuso et al., 2023[296], Marx et al., 2021[305]). KYN and KYN-to-Trp ratio behave more like context markers whose direction and magnitude depend on compartment, medication status, and severity (Almulla et al., 2022[14], Almulla et al., 2022[15], Ou et al., 2023[365]).

When evidence is mapped to symptom modules, clinical meaning tends to sharpen. Reward dysfunction is a clear example (Cléry-Melin et al., 2019[88], Ely et al., 2021[119], Wang et al., 2021[506]). Anhedonia and reduced motivation reflect mesolimbic and frontostriatal circuitry that is sensitive to inflammatory physiology and to KYN-derived neuroactive metabolites (Chen et al., 2021[82], Cooper et al., 2018[91], Haroon et al., 2020[184]). Associations between flux indices and reward-related symptoms often remain detectable even when overall depression severity is controlled, whereas relationships with total depression scores are inconsistent (Chen et al., 2021[82], Cooper et al., 2018[91], Ou et al., 2023[365]). Pooling medicated and unmedicated patients can blur these links, which is one reason seemingly negative studies do not necessarily contradict the broader pattern (Liang et al., 2022[269], Serretti, 2023[432], Zhao et al., 2024[542]). If you look for pathway relationships to a mixture of sadness, guilt, appetite change, sleep change, and psychomotor features, you are asking a single biomarker to explain multiple (Chen et al., 2021[82], Liang et al., 2022[269], Serretti, 2023[432]). If you look for relationships to reward learning, interest, effort expenditure, and motivational drive, the mapping becomes more plausible and coherent (Bustamante et al., 2024[67], Su and Si, 2022[454], Więdłocha et al., 2018[510]). 

A parallel story holds for fatigue and sickness-like dimensions. Data-driven symptom clustering repeatedly shows that elevated inflammatory markers concentrate in tiredness, anergia, and neurovegetative profiles rather than in global severity (Franklyn et al., 2022[149], Milaneschi et al., 2021[323], Moriarity et al., 2023[338]). This matches the biology of sickness behavior, where immune activation recruits coordinated changes in energy allocation, sleep, appetite, and motivation (Dantzer et al., 2008[100], Khandaker et al., 2021[233], Maes et al., 2012[290]). Within depression cohorts, Trp depletion and KYN-to-Trp elevation, when present, often align more closely with fatigue and somatic symptom burden than with mood reactivity (Al-Hakeim et al., 2023[12], Almulla et al., 2022[14], Lanser et al., 2020[255]). This module-first logic also provides a bridge to treatment resistance (Franklyn et al., 2022[149], Lee and Giuliani, 2019[262], Turkheimer et al., 2023[488]). Residual fatigue and anhedonia are among the symptoms most likely to persist despite standard serotonergic strategies, and they are also symptoms most likely to track immune metabolic biology (Chu et al., 2019[87], Frank et al., 2021[148], Milaneschi et al., 2021[322]). 

Cognitive slowing and executive dysfunction represent a third module that often relates to immune burden and to KYN branch balance (Bravi et al., 2025[58], Haroon et al., 2020[184], Savitz, 2020[423]). Cognitive impairment in depression is heterogeneous. Some patients report subjective fog with relatively preserved objective performance, while others show measurable reductions in processing speed and executive control that predict functional disability (Pan et al., 2019[371], Rhee et al., 2024[401], Varghese et al., 2022[498]). Pathway associations tend to be more interpretable when cognition is assessed using objective tasks rather than self-report alone, and when immune context is measured (Knight and Baune, 2018[239], Xi et al., 2024[520], Yang et al., 2025[526]). Both KYNA and QA can plausibly influence glutamatergic signaling and synaptic plasticity, but the clinical interpretation is nuanced (Bertollo et al., 2025[48], de Bartolomeis et al., 2025[101], Savitz, 2020[423]). KYNA is often framed as neuroprotective, yet higher levels can impair cognitive processes through receptor level antagonism and altered cortical signaling (Ostapiuk and Urbanska, 2022[364], Pathak et al., 2024[377], Savitz, 2020[423]). QA is frequently discussed in relation to excitotoxic mechanisms and neuroinflammatory signaling, and central QA is more interpretable than peripheral proxies (Haroon et al., 2020[184], Hestad et al., 2022[192], Savitz, 2020[423]). In practice, the most defensible interpretation is not a single metabolite story but a balance story that needs deeper panels and careful compartment awareness (Haroon et al., 2020[184], Marx et al., 2021[305], Savitz, 2020[423]).

Suicidality is a distinct endpoint rather than simply severe depression. The pathway literature on suicidal ideation and behavior is smaller, but when signals appear, they can look sharper than for total depressive severity (Erhardt et al., 2013[121], Johnston et al., 2022[212], Riera-Serra et al., 2024[403]). This may reflect closer coupling to glutamatergic stress physiology and neuroinflammatory state shifts (Erhardt et al., 2013[121], Gowin et al., 2024[170], Tubbs et al., 2024[486])[453]. Endpoint definition still matters. Ideation, attempt history, and acute crisis states are not equivalent, and timing relative to hospitalization, medication changes, and sleep disruption can move biomarkers (Strumila et al., 2024[453], Sudol and Mann, 2017[455], Wiebenga et al., 2022[508]). The practical implication is that suicidality research benefits from repeated measures and a separation of ideation from behavior, ideally paired with immune indices and, when feasible, central readouts (Bernert et al., 2017[47], Gowin et al., 2024[170], Sudol and Mann, 2017[455]).

### 5.3 Bipolar disorder: phase-aware module map

Bipolar disorder adds a moving target. Phase shifts remodel sleep, energy balance, and immunometabolic labs, and mixed states can appear biologically louder than depression alone (Lyu et al., 2023[286], Mukherjee et al., 2018[342], Rowland et al., 2018[406]). Mood stabilizers and antipsychotics reshape weight, glucose and lipid biology, and inflammatory tone, and they can plausibly modulate Trp to KYN metabolism directly or indirectly (Carli et al., 2021[74], de Melo et al., 2017[107], Kong et al., 2024[240]). Bipolar evidence is therefore best read with phase and treatment exposure treated as primary variables rather than afterthought covariates (Almulla et al., 2022[14], Fellendorf et al., 2022[138], Rowland et al., 2018[406]). 

Across bipolar cohorts, immune markers and metabolic state vary across depressive, manic, and euthymic (Almulla et al., 2022[14], Liu et al., 2018[275], Salagre et al., 2023[410]). The most interpretable pathway findings are those that report phase explicitly and measure enough metabolites to distinguish entry-level catabolism from downstream routing (Almulla et al., 2022[14], Brum et al., 2023[62], Liu et al., 2018[275]). Most studies center on Trp, KYN, and KYN-to-Trp ratio, so the headline signal often reads as Trp down (Bartoli et al., 2021[36], Bravi et al., 2025[58], van den Ameele et al., 2020[492]). When panels extend, findings more often point to altered KYNA and KYNA-based ratios, and some reports suggest QA-to-KYNA imbalance or 3-HK-related indices in mania or bipolar depression, consistent with a possible downstream tilt (Bartoli et al., 2021[36], Birner et al., 2017[53], van den Ameele et al., 2020[492]). Downstream coverage remains inconsistent, and CSF work is limited, so blood-to-brain inference should be made cautiously (Birner et al., 2017[53], Li et al., 2024[268], Marx et al., 2021[305]). 

Module parsing clarifies interpretation. Cognitive efficiency and psychomotor control are durable determinants of functioning in bipolar illness, and they plausibly couple to inflammatory and metabolic load even though individual biomarker studies remain mixed across phases (Keramatian et al., 2021[232], Altamura et al., 2024[17], Van Rheenen et al., 2020[496]). Relapse risk converges on sleep and energy dysregulation, low interest and anergia, and social disability, domains that align with a pathway informed reading in which immune metabolic biology is most informative when mapped onto transdiagnostic modules embedded within bipolar illness rather than averaged mood totals that blend distinct mechanisms (Bai et al., 2026[31], Gitlin and Miklowitz, 2017[164], Harvey, 2008[187]). Translation is constrained by sparse phase stratification, narrow immune phenotyping, and pervasive polypharmacy that can imprint immunometabolic baselines (Berk et al., 2017[46], McIntyre et al., 2022[308], Van Rheenen et al., 2020[496]). 

### 5.4 Schizophrenia spectrum and psychosis: exposure-aware module map

In psychosis, immune signals shift with stage and treatment status, so inflammation is rarely a single story. Early phase psychosis can show innate immune activation before long-term metabolic and lifestyle confounds accumulate, while chronic SCZ cohorts are shaped by medication exposure, smoking, adiposity, and cardiometabolic comorbidity (Howes et al., 2018[199], Michalczyk et al., 2023[320], Upthegrove and Khandaker, 2020[490]). Within this context, the KYN literature has often emphasized KYNA, which makes mechanistic sense if one starts from glutamatergic modulation and cognition, but it can become a single molecule spotlight that obscures branch-level dynamics (Box 1) (Erhardt et al., 2017[124], Sapienza et al., 2023[418], Wonodi and Schwarcz, 2010[514]).

-------

**Box 1: The KYNA paradox is a compartment problem, not a diagnosis fact**

A recurring source of confusion in psychosis is that blood KYNA often trends down or looks inconsistent, while CSF studies more often show KYNA up, and concordance work indicates that KYNA is among the weakest blood-to-CNS proxies compared with KYN or 3-HK.

The practical implication is simple: a “low KYNA” result cannot be read as evidence against central KYNA elevation unless the study actually samples a brain-adjacent compartment or uses a validated bridge.

Treat the paradox as a design signal. It flags matrix choice, state effects, and local KAT-relevant cellular configurations-including astrocytic, peripheral, and potentially mitochondrial aminotransferase contexts-as likely drivers of directionality, not an immutable SCZ signature.

**Test plan (pre-specified)**

- Paired subset: plasma or serum Trp, KYN, 3-HK, KYNA, QA plus CSF KYNA and CSF KYN.
- Prediction: plasma KYN or KYN/Trp tracks CSF KYNA better than plasma KYNA does.
- Moderator: high-cytokine subgrouping and antipsychotic exposure reshape the coupling.

Crucially, the bridge should be branch resolved. Measure QA and 3-HK and report ratios such as KYNA/QA and 3-HK/KYN, alongside immune tone and BBB-relevant markers where feasible. Accordingly, KYNA should be interpreted as a branch-balance readout alongside QA, 3-HK, immune tone, and exposure structure.

-------

Central oriented reports more often support elevated KYNA and higher central KYN-to-Trp ratio, while peripheral case-control studies have sometimes reported lower plasma KYN and KYNA with variable ratio change (Cao et al., 2021[72], Inam et al., 2023[205], Marković et al., 2023[298], Morrens et al., 2020[339], Plitman et al., 2017[390]). Without concurrent measures of entry flux, QA, and key branch intermediates, it is difficult to know whether a KYNA difference reflects true rerouting, treatment and adiposity related remodeling, or compartment mismatch (Box 1) (Kindler et al., 2020[237], Savitz, 2020[423], Skorobogatov et al., 2021[439]). Studies that broaden the panel in minimally treated first-episode psychosis are informative because they can reveal patterns compatible with altered balance rather than isolated shifts (Cao et al., 2021[72], Kuuskmäe et al., 2023[247], Morrens et al., 2020[339]). 

Clinical leverage emerges when pathway measures are paired with anchors that define durable illness burden, especially negative symptoms and cognitive impairment (Al-Hakeim et al., 2020[11], Giordano et al., 2024[163], Kalisova et al., 2023[224]). Positive symptom intensity fluctuates and often responds to dopamine antagonism, while negative and cognitive domains drive functional disability and remain difficult to treat (Correll and Schooler, 2020[94], Giordano et al., 2024[163], Kalisova et al., 2023[224]). Dimensional analyses place experiential deficits such as avolition, anhedonia, and asociality near the center of symptom networks, with social functioning acting as a bridge linking symptom clusters and cognition (Abplanalp et al., 2022[2], Ahmed et al., 2022[7], Cai et al., 2025[70], Charernboon, 2021[78], Giordano et al., 2024[163]). These domains are also where confounding is most dangerous because smoking, adiposity, and antipsychotics influence inflammation, insulin signaling, lipid biology, and Trp availability, and they can also influence cognition directly (Al-Hakeim et al., 2020[11], MacKenzie et al., 2018[288], Toulopoulou et al., 2019[484]). Without careful modeling, pathway phenotype links can be inflated or distorted (Habtewold et al., 2020[177], Maes and Anderson, 2021[289]). 

A realistic stratification strategy in psychosis is therefore stage- and immune-aware (Aymerich et al., 2025[26], Bishop et al., 2022[54], Catalan, 2025[76]). Early-phase cohorts, ideally first-episode or minimally treated samples split by inflammatory burden, provide clean tests of whether KYN signatures flag subgroups with distinct negative symptom or cognitive trajectories (Box 1) (Dunleavy et al., 2022[114], Maes et al., 2020[292], Martinuzzi et al., 2019[302], Mondelli et al., 2015[333], Nettis et al., 2019[352], Toulopoulou et al., 2019[484]). This supports biomarker-enriched add-on trials that target inflammation or immunometabolic risk while treating candidate agents as hypothesis-driven adjuncts rather than universal solutions (Dunleavy et al., 2024[115], Foley É et al., 2023[143], Palmer et al., 2025[370]). Progress is slowed by uneven measurement of smoking and metabolic status and by thin cross-compartment validation (Byrne et al., 2023[69], Catalan, 2025[76]). 

### 5.5 Anxiety and stress-related disorders: module map under thinner evidence

Stress and trauma-related disorders offer a complementary perspective because stress system activation is central and dynamic coupling is, in principle, testable with repeated measures (Dunlop and Wong, 2019[116], Kuzminskaite et al., 2020[249], Sanford et al., 2023[414]). PTSD is mechanistically attractive for Trp to KYN research because sustained HPA and autonomic activation can keep immune signaling above baseline (Dunlop and Wong, 2019[116], Kim et al., 2019[236]). Still, heterogeneity is large. Trauma type and timing, symptom chronicity, sex, medication exposure, smoking, adiposity, cardiometabolic or autoimmune comorbidity, and comorbid depression all reshape inflammatory tone (Lawrence and Scofield, 2024[260], Sanford et al., 2023[414], von Majewski et al., 2023[501]). KYN findings are therefore most interpretable when immune context and comorbidity structure are modeled explicitly, and when symptom modules are separated rather than collapsed into a single total score (Haroon et al., 2020[184], Lamers et al., 2020[253], Milaneschi et al., 2020[324]). 

Most work in this space evaluates entry-level proxies such as Trp, KYN, and KYN-to-Trp ratio, while downstream coverage of KYNA and QA is less consistent (Arnone et al., 2018[22], Haroon et al., 2020[184]). Even so, patterns increasingly look cluster-sensitive (Hunt et al., 2020[203], Kadriu et al., 2021[221]). Some datasets fit a stress-exposed, immune-tuned phenotype with altered aryl hydrocarbon signaling, whereas others suggest that higher entry flux aligns with severe profiles characterized by hyperarousal, sleep disruption, pain, and negative mood (Haroon et al., 2020[184], Jang et al., 2022[209], Tanaka et al., 2021[475]). Chronic stress models add plausibility for downstream imbalances relevant to threat reactivity and impaired extinction (de Bartolomeis et al., 2025[101], Fuertig et al., 2016[152], Kim et al., 2019[236]). The key clinical opportunity is repeated measures designs that test whether within-person immune fluctuations couple to concurrent pathway shifts and symptom oscillations (Haroon et al., 2020[184], Hunt et al., 2020[203], Kadriu et al., 2021[221]). 

Anxiety disorders remain less studied in the KYN literature. Available evidence is best interpreted at the level of stress-linked immune modulation rather than as a uniform diagnostic signature (Evrensel et al., 2020[125], Kim et al., 2019[236], Tanaka et al., 2021[475]). Chronic sympathetic arousal, insomnia, gut permeability, and metabolic status can all nudge baseline inflammation, and these modifiers vary widely between cohorts (Tanaka et al., 2021[475], Więdłocha et al., 2021[509]). As a result, signals look inconsistent across samples that differ in trauma exposure, obesity, alcohol use, or post-infection immune activation (Hunt et al., 2020[203], Kucukkarapinar et al., 2022[246], Tanaka et al., 2021[475]). Hyperarousal and sleep related fatigue likely sit closest to immune-coupled pathway remodeling, while cognitive worry may map less cleanly without standardized modules and sampling around stressors (Fuertig et al., 2016[152], Li et al., 2020[266], Lim et al., 2021[272]). 

### 5.6 Sleep and circadian phenotypes as cross-cutting modulators

Sleep and circadian disruption behave like a volume control for both immune tone and symptom expression (Besedovsky et al., 2019[49], Cox et al., 2022[96], Haspel et al., 2020[188]). They are not just comorbid features. They can reshape pathway interpretation. Sleep loss can elevate inflammatory signaling, alter cortisol dynamics, shift Trp availability, and change energy metabolism (Bhat et al., 2020[51], Garbarino et al., 2021[157], Thompson et al., 2022[481]). Circadian misalignment can decouple symptom reports from biomarker sampling time, so two people with similar biology measured at different circadian phases can look biologically different (Haspel et al., 2020[188], Wilkinson et al., 2019[511], Wright et al., 2015[515]). Module-wise, sleep disturbance intensifies fatigue and sickness-like symptoms, cognitive slowing, emotional reactivity, stress sensitivity, and reward dysfunction (Besedovsky et al., 2019[49], Palmer et al., 2024[369], Thompson et al., 2022[481]). Accordingly, failure to account for sleep and circadian state may lead to misattribution of observed effects to KYN pathway biology, when these are partially driven by sleep-dependent immune and metabolic alterations (Besedovsky et al., 2019[49], Faraut et al., 2022[127], Garbarino et al., 2021[157]). 

### 5.7 Cross-diagnostic synthesis by module

Across diagnoses, the cross-diagnostic synthesis is clearer when framed by modules. Fatigue and sickness-like dimensions show the most stable alignment with immune-coupled KYN activity, particularly when inflammatory burden is elevated and when sampling and metabolic confounds are handled (Hunt et al., 2020[203], Kavyani et al., 2024[228], Li et al., 2020[266]). Cognitive slowing and executive dysfunction show a plausible and often detectable relationship, especially when objective tasks are used and when branch balance is assessed rather than inferred from a single marker (Ahlberg Weidenfors et al., 2025[6], Skorobogatov et al., 2021[439]). Reward and motivational dysfunction also align, particularly when anhedonia is measured as a focused construct rather than absorbed into total symptom totals (Chen et al., 2021[82], Haroon et al., 2020[184], Lucido et al., 2021[283]). Suicidality may yield clinically sharp signals but remains limited by endpoint heterogeneity and sparse central data (Ahlberg Weidenfors et al., 2025[6], Bartoli et al., 2022[35], Skorobogatov et al., 2021[439]). Threat and worry modules in anxiety remain less stable, and the most plausible links appear where hyperarousal, sleep disruption, inflammation, or depressive modules are prominent (Groven et al., 2021[173], Leclercq et al., 2021[261], Tanaka et al., 2021[475]). 

### 5.8 What changes with treatment, regardless of modality

Treatment effects often decouple from pathway normalization, and that decoupling is informative. Patients can improve clinically while pathway indices remain altered if chronic inflammation persists through obesity, autoimmune comorbidity, or smoking, or if sleep and circadian disruption remain untreated (Fishbein et al., 2021[141], Młynarska et al., 2025[330], Pinzi et al., 2025[387]). Conversely, anti-inflammatory interventions can shift biomarkers without immediate symptom relief if downstream circuits remain dysregulated (Goldsmith et al., 2023[166], Pinzi et al., 2025[387], Valizadeh et al., 2025[491]). Baseline inflammation repeatedly predicts who shows measurable pathway change, so biomarker stratification is not a luxury. Outcomes also matter. Module-targeted endpoints frequently reveal more than total scores, because many interventions affect energy, cognition, or motivation disproportionately (Fiszdon et al., 2024[142], Goldsmith et al., 2023[166], van den Heuvel et al., 2025[493]). Longitudinal designs capable of testing mediation, meaning immune change leading to pathway change leading to symptom change, remain underrepresented, and deeper metabolite panels are still needed to resolve branch-level questions (Gygi et al., 2024[176], Hilley and O'Rourke, 2022[193], Zheng et al., 2022[543]). 

### 5.9 Section 5 take-home summary

KYN pathway signals map better to symptom modules than to diagnostic labels, and the mapping becomes strongest when immune state and metabolic context are measured with discipline, when panels are deep enough to test downstream balance, and when designs are longitudinal enough to test temporal ordering. Translation therefore points toward biomarker-enriched trials and toward reporting standards that treat inflammation, sleep, medication exposure, and metabolic status as primary context. That is how the field moves from association to mechanism, and from mechanism to actionable stratification. Association alone cannnot establish directionality; we therefore turn to preclinical causal leverage.

## 6. Preclinical Evidence and Translational Alignment: Measurement, Interpretation, and the Sickness Trap

Preclinical models provide the clearest causal leverage for the Trp-KYN pathway because immune triggers and pathway nodes can be manipulated directly, but translation fails when acute sickness behavior is misread as depression-like behavior, obscuring which effects reflect transient inflammatory malaise versus sustained changes in reward, cognition, or social function (Dantzer, 2017[99], Parrott et al., 2016[375], Savitz, 2020[423]). Throughout this section, diagnoses are treated as context and the readout is module-aligned behavior. Evidence is therefore interpreted through two linked questions: what is mechanistically plausible, and what is mechanistically supported by node-level perturbation, branch-resolving metabolite profiles, and time courses that separate acute from post-acute effects (Table 6(Tab. 6); References in Table 6: Acuña Hidalgo and Armitage, 2022[4]; Bergamini et al., 2018[45]; Chen et al., 2021[82]; Frank et al., 2020[147]; Frenois et al., 2007[150]; Granger et al., 2013[171]; Harden et al., 2006[183]; Lucido et al., 2021[283]; Mauch-Mani et al., 2017[306]; Box 2). We organize the literature by model class-immune challenge, chronic stress, microbiome manipulation, and direct pathway-node perturbation-while asking which designs best support translational alignment with human symptom modules.

### 6.1 Immune challenge models (lipopolysaccharide / polyinosinic:polycytidylic acid, and cytokines) 

Immune challenge models are most informative when treated as sufficiency tests for inflammatory triggers rather than generic models of depression. Their main value is that they permit controlled induction of cytokine-linked Trp diversion and therefore make timing visible: early windows are dominated by sickness motivation, thermoregulation, reduced locomotion, and entry-level Trp-KYN changes, whereas later windows are more informative for reward, cognition, social interaction, and branch-resolving metabolite patterns (Table 6(Tab. 6)) (Dantzer, 2001[98], Moreau et al., 2008[337]). This time dependence is not a technical footnote; it determines what the behavior means. A reduced score during the acute cytokine peak may index malaise, whereas persistent changes after the acute window can support stronger inference about psychiatric-relevant modules (Bay-Richter et al., 2011[39], Hunt et al., 2020[203], Moreau et al., 2008[337]). Interpretable studies therefore align behavioral testing with biological phase rather than treating all post-challenge time points as equivalent (Lasselin et al., 2020[258], Moreau et al., 2008[337], Tchessalova et al., 2018[479]). 

This distinction matters because the dominant biology shifts across the post-challenge trajectory. During the acute phase, the animal is negotiating a real neuroimmune state characterized by reduced exploration, lower food intake, altered temperature regulation, and changes in general activity (Dantzer, 2001[98], Kelley et al., 2003[230], Lasselin et al., 2020[258]). Those effects should be understood as sickness, not repackaged as depression-like behavior simply because they lower task output (Dantzer, 2001[98], Kelley et al., 2003[230], Moreau et al., 2008[337]). The post-acute window is more useful for asking whether reward processing, effort expenditure, memory, or social engagement remain altered after the acute cytokine surge has started to resolve (Bay-Richter et al., 2011[39], Harrison et al., 2016[186], Tchessalova et al., 2018[479]). Later persistence windows can be even more informative if the design includes repeated measures or second-hit logic (Carlezon et al., 2019[73], Moreau et al., 2008[337], Turano et al., 2021[487]). Without that temporal structure, single-point assays can easily overstate behavioral specificity.

The main interpretive hazard in this literature is the sickness trap. Early locomotor suppression, reduced exploration, and altered feeding are genuine neuroimmune effects, but they should not be relabeled as depression-like behavior without motor and physiological controls (Bay-Richter et al., 2011[39], Dantzer, 2001[98], Kelley et al., 2003[230]). Forced-swim or tail-suspension outcomes during high-sickness windows are especially vulnerable to overinterpretation, as are social readouts that depend heavily on intact locomotion (Frenois et al., 2007[150], Lasselin et al., 2020[258], Moreau et al., 2008[337]). More persuasive immune-challenge studies pair module-relevant tasks with intake or motor controls, include repeated sampling across acute and post-acute windows, and measure branch markers rather than relying on Trp, KYN, or KYN/Trp ratio alone (Hunt et al., 2020[203], Moreau et al., 2008[337], Zhang et al., 2024[540]). The practical rule is simple: acute sickness should be treated as state, not phenotype (Table 6(Tab. 6)).

### 6.2 Chronic stress models (chronic unpredictable mild stress, social defeat, and chronic variable stress)

Chronic stress models offer a different kind of translational value because they probe sustained neuroendocrine-immune coupling rather than an acute inflammatory pulse (de Bartolomeis et al., 2025[101], Hassamal, 2023[189], Tong et al., 2023[482]). That makes them conceptually closer to the LGI background relevant to psychiatry, especially when stress exposure produces altered glucocorticoid signaling, immune activation, and persistent changes in Trp routing without a large acute cytokine surge (Tanaka et al., 2021[475], Xu et al., 2025[521]). Their advantage is ecological persistence; their limitation is interpretive dispersion. Because stress paradigms vary widely in duration, intensity, controllability, sex effects, baseline physiology, and behavioral readouts, they can generate broad phenotypes without clearly locating where the pathway is being engaged (Bergamini et al., 2018[45], Tong et al., 2023[482]).

For that reason, chronic stress models are most persuasive when they move beyond headline KYN/Trp ratio changes and include branch-resolving metabolites, inflammatory context, and module-focused behavior such as effort-based reward, cognition, social interaction, or stress reactivity (Bergamini et al., 2018[45], Fuertig et al., 2016[152]). Used this way, they are valuable not as disease replicas, but as models of sustained context in which endocrine and immune drivers can jointly bias pathway routing over time (Deng et al., 2021[109], Tanaka et al., 2021[475]). They are especially useful for asking whether prolonged stress exposure produces a stable biochemical milieu that resembles the human background field described in Section 3, rather than only a short-lived inflammatory perturbation (Luo et al., 2025[284], Ye et al., 2024[529]).

At the same time, chronic stress models can become too behaviorally broad if pathway interpretation is not disciplined (Bergamini et al., 2018[45], de Bartolomeis et al., 2025[101]). An effect on general activity, for example, is much less informative than a selective change in reward pursuit with preserved motor ability, or a memory deficit accompanied by a branch-resolving KYN signature (Bergamini et al., 2018[45], Fuertig et al., 2016[152], Li et al., 2023[264]). The most convincing studies therefore combine chronic stress exposure with repeated biological sampling, explicit behavioral module mapping, and enough pathway depth to distinguish entry effects from downstream rerouting (Deng et al., 2021[109], Fuertig et al., 2016[152]). Without that structure, stress models can remain plausible but mechanistically underdetermined (Table 7(Tab. 7); References in Table 7: Alexander et al., 2012[10]; Erhardt et al., 2017[123]; Foster et al., 2021[144]; Kealy et al., 2020[229]; Kindler et al., 2020[237]; Kozak et al., 2014[242]; Kubota et al., 2022[245]; Lasselin, 2021[257]; Liu et al., 2018[277]; Markov, 2022[297]; Ou et al., 2023[365]; Potter et al., 2010[392]; Primo et al., 2023[393]) (Hassamal, 2023[189]).

### 6.3 Microbiome manipulations (psychiatric-relevant slice)

Microbiome manipulation studies are most useful for this review when they assess psychiatric-relevant behaviors and measure Trp-KYN outputs at the same time, because microbiota-driven shifts in Trp routing can influence immune tone and precursor availability without necessarily producing a fully characterized downstream pathway signature (Agus et al., 2018[5], Kennedy et al., 2017[231], Zhu et al., 2020[545]). The most interpretable designs quantify both serotonin-branch and KYN-branch indices and report behavior in reward, threat, cognition, or social domains rather than broad health phenotypes (Deng et al., 2021[109], Zhou et al., 2023[544]). A persistent limitation is incomplete metabolite coverage, which makes it difficult to distinguish true branch rebalancing from upstream precursor effects, and the mechanistic locus often remains ambiguous because microbiome shifts alter multiple immune and metabolic pathways at once (Agus et al., 2018[5], Gao et al., 2018[156], Leclercq et al., 2021[261]).

Given these constraints, microbiome work is best viewed as an upstream-routing stream rather than the strongest causal test of pathway decisions (Box 2) (Deng et al., 2021[109], Hou et al., 2023[198], Kennedy et al., 2017[231]). It can show that ecological perturbation of the host environment shifts Trp availability, inflammatory tone, and behavior together, but it rarely isolates which biochemical node is responsible unless combined with deeper pathway measurement or direct perturbation logic (Cheng et al., 2023[85], Deng et al., 2021[109], Leclercq et al., 2021[261]). In translational terms, microbiome studies are useful for identifying plausible upstream architecture, but they are less definitive than node-level interventions for establishing causal pathway leverage (Agus et al., 2018[5], Zhu et al., 2020[545]). 

### 6.4 Direct manipulation of kynurenine pathway nodes (IDO/TDO/KMO/KAT)

**Figure 4:**
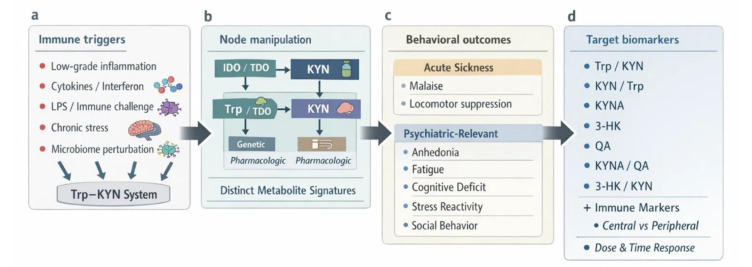
Translational dissection of the Trp-KYN pathway: linking immune triggers, node-specific manipulation, behavioral phenotypes, and target-engagement biomarkers. Schematic overview of a translational pipeline connecting upstream immune or stress-related triggers to node-specific manipulation of the Trp-KYN pathway and downstream behavioral readouts. Entry-point enzymes and branch-routing nodes are where the preclinical literature becomes most clinically relevant. If a study claims that altering KMO changes reward behavior, the question is not simply whether reward behavior moved, but whether 3-HK- and QA-related biology shifted in the expected direction and whether the behavioral effect remained once nonspecific sickness or sedation was excluded. Likewise, if KAT modulation is proposed to influence cognition or salience processing, the study should demonstrate a corresponding KYNA-related signature rather than inferring branch change from a single upstream ratio. The stronger the biochemical prediction, the stronger the translational inference. 3-HK, 3-hydroxykynurenine; KAT, kynurenine aminotransferase; KMO, kynurenine 3-monooxygenase; KYN, kynurenine; KYNA, kynurenic acid; QA, quinolinic acid; Trp, tryptophan.

Given these limitations, the strongest causal stream remains direct perturbation of pathway nodes, where target engagement and downstream effects can be quantified explicitly (Box 2). Genetic models and pharmacologic modulation of IDO, TDO, KMO, or KAT provide the clearest test of whether altering a defined biochemical decision point is sufficient to shift metabolite profiles and modify reward, cognition, social behavior, or stress reactivity (Notarangelo and Pocivavsek, 2017[354], Pocivavsek et al., 2024[391], Szabó et al., 2025[458]). This stream matters most for translation because it converts pathway biology into testable intervention logic: node selection implies a predicted metabolite signature, which should be demonstrable in a dose- and compartment-aware manner (Figure 4(Fig. 4)) (Platten et al., 2019[389], Song et al., 2017[446]). A persuasive node-manipulation study therefore does more than alter behavior; it shows that the expected biochemical rerouting occurred.

To be translationally persuasive, node-manipulation studies must also show that behavioral effects are not secondary to nonspecific sickness, sedation, or locomotor suppression (Lim et al., 2021[272], Schettino et al., 2024[425]). The strongest designs combine an immune trigger with node manipulation and regional metabolite readouts, allowing early hypoactivity to be separated from later changes in reward, cognition, or social behavior (Notarangelo and Pocivavsek, 2017[354], Savitz, 2020[423], Szabó et al., 2025[458][459]). This is the practical antidote to the sickness trap: the manipulation is interpreted through time-locked target engagement plus module-relevant behavior, not through a single assay read at the wrong phase (Lim et al., 2021[272], Savitz, 2020[423], Schettino et al., 2024[425]).

-------

**Box 2: Translational-weight checklist for preclinical Trp-KYN studies**

*Use as a rapid scoring rubric to judge how strongly a preclinical study supports translational inference.*

**1. Defined trigger: **Immune challenge (LPS/Poly(I:C)/cytokine), chronic stress, microbiome manipulation, or node perturbation is specified with dose, route, and timing.

**2. Branch-resolving Trp-KYN panel: **At minimum includes Trp, KYN, KYNA, 3-HK, QA, or validated branch ratios such as KYNA/QA and 3-HK/KYN.

**3. Module-aligned behavior: **Outcomes map onto reward/anhedonia, fatigue/energy regulation, cognition, social withdrawal, or threat/hyperarousal rather than relying on a single “despair” assay.

**4. Target engagement: **Enzyme manipulation (genetic or pharmacologic) is paired with metabolite shifts consistent with the targeted node; dose-response is preferred.

**5. Time-course separation: **The design explicitly separates the acute sickness window from post-acute or persistent behavioral changes.

---

**Interpretation: **0-3 = low translational weight; 4-7 = moderate translational weight; 8-10 = high translational weight.

-------

#### 6.4.1 Target engagement logic: what must be shown

A target-engagement lens clarifies what good Trp-KYN manipulation studies look like (Figure 4(Fig. 4)). The intervention should shift a pre-specified metabolite pattern consistent with the targeted node, ideally in dose-response form, while behavioral changes should map onto modules and be separable from locomotor suppression or acute inflammatory malaise (Bansal et al., 2022[33], Parrott et al., 2016[375], Pocivavsek et al., 2024[391]). Branch markers are not optional. KMO modulation should move 3-HK- and QA-related indices, KAT-directed modulation should shift KYNA-related indices in an isoform- and compartment-consistent manner (Table 2(Tab. 2)), and upstream IDO or TDO interventions should be interpreted using entry and branch readouts together rather than relying on KYN/Trp ratio alone (Amori et al., 2009[19], Badawy, 2017[27], Bai et al., 2021[32], Szabó et al., 2025[458][459]). For KAT-directed studies, an additional layer of rigor is needed because not all KAT manipulations are mechanistically equivalent. A putative brain-directed KYNA intervention is most interpretable when the relevant isoform and compartment of engagement are specified, or at minimum when regional or brain-adjacent metabolite data support a cerebral mechanism (Bai et al., 2021[32], Pocivavsek et al., 2024[391], Sathyasaikumar et al., 2011[421], Tanaka et al., 2026[467]). By contrast, a generic “KAT effect” observed in blood or whole tissue may reflect a different aminotransferase context with different implications for behavior, cognition, or inflammatory coupling (Bai et al., 2021[32], Moulin et al., 2024[341], Rossi et al., 2019[405]). Isoform-aware target engagement would therefore strengthen the translational logic of KAT studies and reduce the risk of overgeneralizing peripheral KYNA shifts as evidence of central pathway modulation (Bai et al., 2021[32], Pocivavsek et al., 2024[391], Sathyasaikumar et al., 2011[421], Tanaka et al., 2026[467]). Central versus peripheral engagement should be separated whenever feasible, since some metabolites dissociate across compartments (Table 7(Tab. 7)).

The deeper principle is that target engagement, compartment awareness, and module-relevant behavior must all line up if a study wants to claim causal pathway relevance. A drug or knockout that changes behavior without changing the predicted metabolite pattern is difficult to interpret (Beaumont et al., 2016[41], Erhardt et al., 2017[123], Parrott et al., 2016[375]). Conversely, a clear biochemical shift without behavioral specificity may indicate pathway engagement without psychiatric relevance (Beaumont et al., 2016[41], Erhardt et al., 2017[123], Parrott et al., 2016[375]). The translational sweet spot lies where biochemistry and behavior converge in the same temporal frame. That is also why repeated sampling is so important: it distinguishes early physiological disruption from later behavioral persistence and makes it possible to ask whether the metabolite pattern precedes, accompanies, or outlasts the behavioral signal (Beaumont et al., 2016[41], Parrott et al., 2016[375], Tanaka and Vécsei, 2025[476]).

This same target-engagement logic is what the clinical literature often lacks, which is why an explicit translational alignment matrix remains useful for connecting human outcomes to animal readouts (Table 7(Tab. 7); Box 2) (Brown et al., 2024[61], Pocivavsek et al., 2024[391], Tanaka and Vécsei, 2025[476]). Preclinical studies can therefore serve as a design template for human work: define the node, predict the metabolite pattern, specify the behavioral module, exclude nonspecific sickness, and interpret central versus peripheral readouts deliberately rather than interchangeably.

### 6.5 Synthesis: what preclinical evidence adds beyond clinical association

Across model classes, preclinical evidence contributes in complementary ways. Immune challenges test sufficiency of inflammatory triggers, stress paradigms probe sustained neuroendocrine-immune coupling, microbiome manipulations test upstream Trp-routing effects, and node perturbations test whether pathway decisions causally shape module-aligned behaviors. The interpretive hazard is consistent across all classes and should be treated as a design variable rather than a rhetorical caveat: acute sickness is a real neuroimmune state, but it is not equivalent to sustained psychiatric-relevant modules (Table 6(Tab. 6)).

These relationships can be summarized as a pipeline from trigger to node to metabolite signature to behavior, an organizing framework that highlights where target-engagement biomarkers are essential for translation (Figure 4(Fig. 4)). Preclinical evidence therefore adds something clinical association cannot: it shows which pathway manipulations are sufficient, which readouts are time-sensitive, and where causal leverage is strongest. It also clarifies what better clinical studies should look like. If human work is to move beyond association, it will need deeper metabolite panels, repeated sampling, more explicit symptom-module mapping, and a clearer commitment to the same target-engagement logic that makes the strongest animal studies persuasive.

## 7. An Interpretation Algorithm for ‘Mixed’ Tryptophan–Kynurenine Findings

Use this section as a decision tool rather than as another narrative review. For any study, first classify immune context and the likely pathway entry driver, then ask whether the metabolite panel resolves branch balance, whether the sampled compartment can support the intended inference, and whether outcomes map to symptom modules rather than diagnosis totals. The goal is not to force convergence where biology is genuinely context-dependent, but to make divergence interpretable and to identify when a null or “mixed” result is the expected consequence of shallow panels, mismatched matrices, or dominant confounding.

### 7.1 Step 1: Is inflammatory tone elevated, and is it measured in a comparable way?

Start with immune context. Evidence can come from CRP, a cytokine pattern, neopterin-like immune activation markers, or a defined immune trigger. If immune tone is not measured, the cohort should not be treated as “immune-low” by default. If inflammatory tone is elevated, Step 1 shifts in Trp and KYN-related indices become more plausible, and the next task is to distinguish entry diversion from downstream branch dominance rather than treating one ratio as the whole story.

### 7.2 Step 2: Entry diversion is not a mechanism claim: IDO-like versus TDO-like context 

A higher KYN/Trp ratio signal is best treated as a marker of increased entry-level diversion, not as proof of a specific enzyme or a uniquely immune mechanism. Non-immune entry drivers matter because KYN/Trp ratio can rise without a classic cytokine signature. Cortisol and other endocrine signals can increase TDO-weighted conversion, while stress-related metabolic drift can alter free Trp availability and amplify the same ratio. Mixed states are therefore expected rather than exceptional: LGI can coexist with HPA-axis activation, and each can bias entry through different gatekeepers. The practical rule is to read KYN/Trp ratio as an entry signal whose upstream driver must be inferred from context, not assumed from the ratio itself. The same entry-level pattern can therefore be generated by different upstream drivers and feed into different downstream branch profiles (Table 4(Tab. 4)).

### 7.3 Step 3: Branch resolution determines whether mechanistic interpretation is licensed

Next ask whether the panel actually resolves branch routing. If branch markers are absent, branch claims should be avoided. Trp, KYN, and KYN/Trp ratio can show that entry diversion is more or less engaged, but they cannot determine whether the pathway is leaning toward KYNA-facing modulation or toward KMO-linked 3-HK and QA biology. At minimum, branch interpretation requires downstream metabolites that make competition between routes visible (Table 5(Tab. 5)). Without them, apparently mixed findings are often not contradictory at all; they are simply underresolved.

### 7.4 Step 4: Compartment logic: blood, cerebrospinal fluid (CSF), and ex vivo immune cells answer different questions

Then ask whether the sampled compartment can support the claim being made. Blood-based studies can be informative for peripheral catabolic engagement and, in some cases, for KYN availability, but they are weaker tools for inferring central branch balance, especially when conclusions rely on KYNA alone. If the matrix is blood-only, central KYNA-facing versus QA-facing interpretation should be downgraded unless complementary evidence exists. Compartment is therefore not a technical footnote; it is part of the causal meaning of the result.

### 7.5 Step 5: Module-first outcomes: map kynurenine (KYN) metabolite patterns to dimensions, not diagnosis totals

Outcome structure matters just as much as biomarker depth. If the readout is a broad diagnosis total, true immune-Trp-KYN effects may be diluted across heterogeneous symptom clusters. In contrast, module-focused outcomes such as fatigue, anhedonia, cognitive dysfunction, negative symptoms, or threat-related stress reactivity are more likely to align with pathway-relevant biology. The practical rule is that diagnosis can define the cohort, but symptom modules should define the biological question whenever the aim is mechanistic interpretation.

### 7.6 Step 6: Confounding structure can dominate and flip apparent case-control differences

Finally, ask which confounders are strong enough to dominate the signal. Smoking, adiposity and metabolic drift, sleep and circadian disruption, recent infection or vaccination, and medication exposures can each shift baseline inflammatory tone and Trp-KYN metabolism. In psychosis cohorts, antipsychotics, smoking prevalence, and metabolic change are often strong enough to overwhelm diagnosis-level signals unless they are exposure-balanced, stratified, or explicitly modeled. When these confounders differ across studies, divergent biomarker directions are not surprising and should be treated as a comparability problem first.

### 7.7 The “If X and Y then Z” rule block: expected patterns under common design states

Use the following rules as a minimal forecast of what a study is likely to show. If immune tone is elevated and the panel is entry-only, then Step 1 diversion is the main inference and branch claims should be avoided. If immune tone is elevated and branch markers are measured, then subgroup-specific branch tilt is more plausible than a single diagnostic signature. If immune phenotyping is weak but stress or endocrine load is high, similar entry signals can arise through non-immune routes and downstream patterns should be expected to vary (Table 4(Tab. 4)). If the sampled compartment is blood-only, central branch balance should not be inferred from peripheral KYNA alone. If outcomes are diagnosis totals rather than modules, true immune-linked Trp-KYN effects are likely to be diluted. If psychosis cohorts are heavily antipsychotic-exposed, smoking-unmatched, or metabolically drifted, exposure architecture is often a stronger predictor of Trp-KYN patterns than diagnosis. If longitudinal Trp-KYN change tracks module change, mechanistic support strengthens even when baseline case-control differences are small.

### 7.8 Worked examples: why two studies can both be “right”

Applied to immune-challenge or medically inflamed contexts, Step 1 diversion and predictable time-locked shifts are expected, and symptom change often aligns with sickness-like dimensions. Applied to spontaneous psychiatric cohorts with heterogeneous immune tone and incomplete panels, the expected outcome is a mixture of weak entry signals and unstable downstream patterns. In psychosis, improving comparators by stage, smoking, BMI, and medication exposure should reduce apparent contradictions and help clarify whether KYNA- or QA-related findings reflect trait-like vulnerability, state change, or exposure-driven biology.

### 7.9 Take-home message

Apparent inconsistency in psychiatric Trp-KYN findings is often more structured than it first appears. Once immune context, entry diversion, branch depth, compartment, outcome choice, and confounding structure are specified, many “mixed” findings become predictable rather than chaotic. The practical implication is simple: better interpretation begins by treating divergence as a design problem before treating it as biological contradiction.

## 8. Research Gaps: Prioritized and Specific (Falsifiable Format)

The field does not mainly suffer from a lack of plausible biology; it suffers from a lack of studies designed to decide among plausible explanations. The most useful next step is therefore not a generic call for more work, but a prioritized agenda built around measurable, correctable gaps. Earlier sections of this review show that many “mixed” findings become more coherent once branch depth, inflammatory context, compartment, timing, and confounding are specified. Section 8 converts that logic into a falsifiable roadmap. Each gap below is framed as a concrete problem, followed by a near-term fix, a longer-term solution, and a success criterion that would show the field has actually moved. The order is intentional: the first gaps are the ones most likely to improve comparability quickly, while the later gaps matter most for mechanistic precision and translation.

### Gap 1. Panels remain too shallow for branch inference

A large share of psychiatric Trp-KYN studies still relies on Trp, KYN, and KYN/Trp ratio alone (Almulla et al., 2022[14], Ou et al., 2023[365], Skorobogatov et al., 2021[439]). That is enough to detect entry-level diversion, but not enough to decide whether the biologically relevant signal lies in KYNA-facing modulation, in KMO-linked 3-HK/QA routing, or in a mixed and time-dependent state (Marx et al., 2021[305], Ostapiuk and Urbanska, 2022[364], Ou et al., 2023[365]). If entry-only panels dominate, the field will continue to mistake underresolution for contradiction (Almulla et al., 2022[14], Ou et al., 2023[365], Skorobogatov et al., 2021[439]). This is a falsifiable gap because studies with deeper panels should produce more interpretable subgroup structure and fewer apparent disagreements than studies restricted to the core trio (Marx et al., 2021[305], Ou et al., 2023[365], Walpole and Newell, 2024[503]). 

Near-term fix: Make branch-resolving panels the default minimum when mechanistic claims are made, including at least KYNA, 3-HK, and QA alongside Trp, KYN, and KYN/Trp ratio.

Long-term fix: Build harmonized multi-analyte panels that can be deployed across cohorts, paired with pre-registered branch hypotheses rather than post hoc ratio fishing.

Success criterion: Across independent cohorts, studies with branch-resolving panels should show clearer convergence in subgroup patterns than studies using entry-only markers.

### Gap 2. Inflammatory phenotyping is still too weak and too inconsistent

Many studies treat diagnosis status as a proxy for inflammatory tone or classify inflammation using non-comparable thresholds and sparse markers (Brinn and Stone, 2020[60], Pedraz-Petrozzi et al., 2020[381], Zainal and Newman, 2021[536]). That weakens inference at the first decision point. If inflammatory context is not measured directly, a cohort cannot be interpreted confidently as immune-high, immune-low, or immunologically mixed (Byrne et al., 2022[68], Pedraz-Petrozzi et al., 2020[381]). This matters because the same KYN/Trp ratio pattern can arise from distinct upstream states. The gap is falsifiable because stricter inflammatory phenotyping should improve comparability across studies and reduce unexplained heterogeneity.

Near-term fix: Require explicit inflammatory characterization using CRP and, where possible, complementary cytokine or immune-activation markers, with thresholds defined before analysis.

Long-term fix: Establish field-wide inflammatory strata that are portable across psychiatric cohorts and compatible with metabolite-based pathway models.

Success criterion: Studies using comparable inflammatory phenotyping should show more stable Trp-KYN associations than diagnosis-only studies, particularly in symptom-module analyses.

### Gap 3. Confounder capture remains too weak for believable psychiatric inference

The main confounders are not mysterious. Smoking, adiposity and metabolic burden, sleep and circadian disruption, medication exposure, renal function, and recent infection or vaccination are all predictable sources of variation in inflammatory tone and Trp-KYN biology (Aarsland et al., 2022[1], Cussotto et al., 2020[97], Hunt et al., 2020[203]). Yet they are still captured unevenly, modeled inconsistently, or acknowledged only after discordant results appear. This makes many psychiatric biomarker papers difficult to compare even before the biology is considered (Coppens et al., 2022[92], Inam et al., 2023[205], Tanaka et al., 2021[475]). The gap is falsifiable because stronger confounder capture should reduce between-study volatility and make exposure architecture more informative than crude diagnosis labels.

Near-term fix: Treat a minimum adjustment set as mandatory rather than optional, with transparent reporting of the variables most likely to dominate the signal.

Long-term fix: Standardize exposure and confounder modules that can be embedded routinely into psychiatric biomarker studies across diagnoses and settings.

Success criterion: After adjustment for the minimum confounder set, diagnosis-linked differences should become smaller but more reproducible, while module-linked associations should become stronger and more stable.

Because these confounders are predictable, the field also needs a simple reproducibility framework that makes omissions obvious and corrections routine rather than aspirational (Box 3).

-------

**Box 3: Top 10 reproducibility killers (and how to fix them)**

*Use this as a rapid manuscript-side checklist before interpreting any psychiatric Trp-KYN result.*

**1. Shallow panels **→ use a minimum branch-resolving panel plus ratios.

**2. Fasting/time-of-day ignored **→ prespecify the sampling window.

**3. BMI without waist **→ record both, plus metabolic markers when feasible.

**4. Smoking unbalanced **→ match or stratify, and quantify exposure.

**5. Medication exposure vague **→ report class, dose, duration, and recent changes.

**6. Infection timing absent **→ document recent illness, vaccination, and antibiotics.

**7. Platform QC missing **→ report calibration, CVs, LLOQs, and batch strategy.

**8. Sample handling drift **→ report tube type, processing delay, storage, and freeze-thaws.

**9. Illness phase pooled **→ define episode, remission, stage, and duration.

**10. Totals over modules **→ preregister module outcomes and the analytic plan.

Interpretive rule: when several of these failures cluster in the same study, apparent psychiatric specificity should be downgraded until the design is repaired.

-------

### Gap 4. Peripheral-to-central inference remains under-validated

Psychiatric interpretation often depends on claims about central branch balance, yet most available studies measure blood alone (Bartoli et al., 2021[36], Coppens et al., 2022[92], Morrens et al., 2020[339]). That is not inherently invalid, but it becomes problematic when peripheral signals are treated as though they transparently mirror brain-adjacent chemistry. KYN and QA may show some cross-compartment alignment under specific conditions, whereas KYNA is much less reliable as a blood-based proxy of central balance (Orhan et al., 2024[360], Paul et al., 2022[379], Skorobogatov et al., 2021[439]). This gap is falsifiable because matched peripheral-central studies should reveal which analytes and ratios travel well across compartments and which do not.

Near-term fix: Avoid central claims from blood-only studies unless the claim is explicitly limited to peripheral pathway engagement or supported by convergent evidence.

Long-term fix: Build cross-compartment datasets linking blood, CSF, imaging, and where possible cellular or tissue-adjacent measures within the same participants.

Success criterion: The field should be able to specify, with evidence, which metabolites are valid peripheral sentinels of central processes and which require direct central measurement.

### Gap 5. Longitudinal and intervention designs are still too rare

Most psychiatric Trp-KYN studies are cross-sectional, which makes them useful for pattern recognition but weak for directionality, mediation, and treatment relevance (Marx et al., 2021[305], Ou et al., 2023[365], Sapienza et al., 2024[416]). A cross-sectional case-control design cannot determine whether inflammation precedes pathway change, whether pathway change precedes symptom change, or whether all three are being moved by the same confounder. This gap is falsifiable because repeated-measures and intervention designs should reveal whether pathway shifts track module change within individuals rather than only between groups.

Near-term fix: Add repeated sampling to observational studies, especially across acute-state resolution, treatment response, or symptom fluctuations in high-yield modules such as fatigue and anhedonia.

Long-term fix: Prioritize longitudinal mediation designs and intervention studies that test whether changing inflammatory or metabolic context shifts branch-resolving metabolites and symptom modules together.

Success criterion: Within-person studies should demonstrate whether module improvement can occur with, precede, or lag behind Trp-KYN normalization, thereby clarifying causal order rather than merely describing association.

### Gap 6. Translational alignment is still too weak

Human and preclinical studies often speak to the same pathway but not to the same biological question. In animal work, causal leverage is strongest when timing, target engagement, and module-relevant behavior are aligned (de Bartolomeis et al., 2025[101], Giménez-Gómez et al., 2021[162], van der Horn et al., 2026[495]). In human work, conclusions are often drawn from shallow panels, poorly defined inflammatory context, and broad diagnosis totals (Gáspár et al., 2021[159], Hunt et al., 2020[203], Lim et al., 2021[272]). The result is a translational mismatch: the preclinical literature may show that a node manipulation shifts a predicted metabolite signature and a specific behavioral module, while the clinical literature measures only KYN/Trp ratio and a total symptom score (Giménez-Gómez et al., 2021[162], Hunt et al., 2020[203], van der Horn et al., 2026[495]). This gap is falsifiable because tighter alignment should improve the ability to compare preclinical and clinical signals directly.

Near-term fix: Align human and animal studies around shared modules, shared branch-resolving biomarkers, and explicit target-engagement logic.

Long-term fix: Develop translational pipelines in which trigger, node, metabolite signature, and module-relevant outcome are specified in parallel across species.

Success criterion: A convincing translational study should be able to connect a defined preclinical node perturbation, a predicted metabolite pattern, and a homologous human symptom module within the same mechanistic framework.

Taken together, these gaps define a ranked agenda rather than a loose wish list. The fastest gains are likely to come from deeper panels, standardized inflammatory phenotyping, and mandatory confounder capture, because those changes can improve interpretability almost immediately. The next major advance will come from cross-compartment validation and repeated-measures designs, which can clarify what blood can and cannot stand in for and whether pathway shifts truly track symptom change. The hardest but most consequential step is stronger translational alignment, because that is what turns association into intervention logic. If the field succeeds on these fronts, the likely result will not be perfect uniformity. Biology will remain context dependent. But the literature should become less noisy, more comparable, and more capable of supporting stratification, target engagement, and falsifiable therapeutic hypotheses. That is the standard by which progress should be judged.

## 9. Conclusions

Across psychiatric disorders, the Trp-KYN pathway remains one of the most plausible biochemical bridges between immune activation, chronic LGI, and symptom expression, but current findings are more informative as a framework than as a standalone clinical biomarker. The biology is compelling because immune and stress-linked signals can reroute Trp toward neuroactive KYNs, and branch balance can, in principle, connect inflammatory tone to reward, energy, and cognitive modules. What blocks clinical readiness is not that the signal is absent, but that studies too often speak different measurement languages. Shallow panels that stop at Trp and KYN, inconsistent immune phenotyping, uneven control of adiposity, smoking, sleep, infection timing, and medication exposure, plus outcomes anchored to diagnosis totals all erode comparability and inflate apparent contradictions (Skorobogatov et al., 2021[439]). 

Reproducibility can improve within the next few years if Trp-KYN studies stop treating design hygiene as optional. Adopt a shared minimum Trp-KYN panel that resolves branch balance, not just entry diversion: Trp, KYN, KYNA, QA, and 3-HK with KYN/Trp, KYNA/QA, and 3-HK/KYN ratios, paired with CRP plus a feasible cytokine cue. Lock sampling and reporting to a checklist, including fasting and clock time, BMI and waist, smoking, medications, sleep, and recent infection timing, since these factors can flip signals across cohorts. Finally, preregister module-first outcomes such as anhedonia, fatigue, cognition, and negative symptoms, and analyze them within CRP-high versus CRP-low strata or immune clusters to turn heterogeneity into branch-specific predictions (Badawy and Guillemin, 2019[30]). 

Long-term progress will come from cell-resolved mapping of Trp-KYN regulation, longitudinal causal modeling that tests immune shifts, Trp-KYN routing, and symptom-module dynamics, and validated target-engagement biomarkers that confirm pathway modulation in humans. With those pieces, precision trials can recruit inflammation- and Trp-KYN-defined subgroups, adapt dosing to biomarker response, and judge success by engagement-linked improvement rather than global score changes. If these priorities are met, Trp-KYN biology can move from a compelling neuroimmune narrative to a practical stratification tool that guides intervention selection in TRD and psychosis (Pocivavsek et al., 2024[391]).

## Notes

Masaru Tanaka and László Vécsei contributed equally as first author.

Masaru Tanaka and László Vécsei (Department of Neurology, Albert Szent-Györgyi Medical School, University of Szeged, H-6725 Szeged, Hungary; Tel.: +36-62-545-351, E-mail: vecsei.laszlo@med.u-szeged.hu) contributed equally as corresponding author.

## Declaration

### Acknowledgments

This work was supported by the National Research, Development, and Innovation Office-NKFIH K138125, SZTE SZAOK-KKA No. 2022/5S729, and the HUN-REN Hungarian Research Network.

### Conflict of interest

The authors declare no conflicts of interest.

### Authors' contribution

Conceptualization, M.T. and L.V.; methodology, M.T.; software, M.T.; validation, M.T. and L.V.; formal analysis, M.T.; investigation, M.T.; resources, M.T.; data curation, M.T.; writing-original draft preparation, M.T.; writing-review and editing, M.T. and L.V.; visualization, M.T.; supervision, M.T. and L.V.; project administration, M.T. and L.V.; funding acquisition, L.V. All authors have read and agreed to the published version of the manuscript.

### Using Artificial Intelligence (AI)

The authors acknowledge limited use of AI during manuscript preparation for language refinement, reference search, preliminary searches, and initial figure design. All outputs were independently reviewed, substantially modified where needed, and approved by the authors, who take full responsibility for the final content of this publication.

## Figures and Tables

**Table 1:**
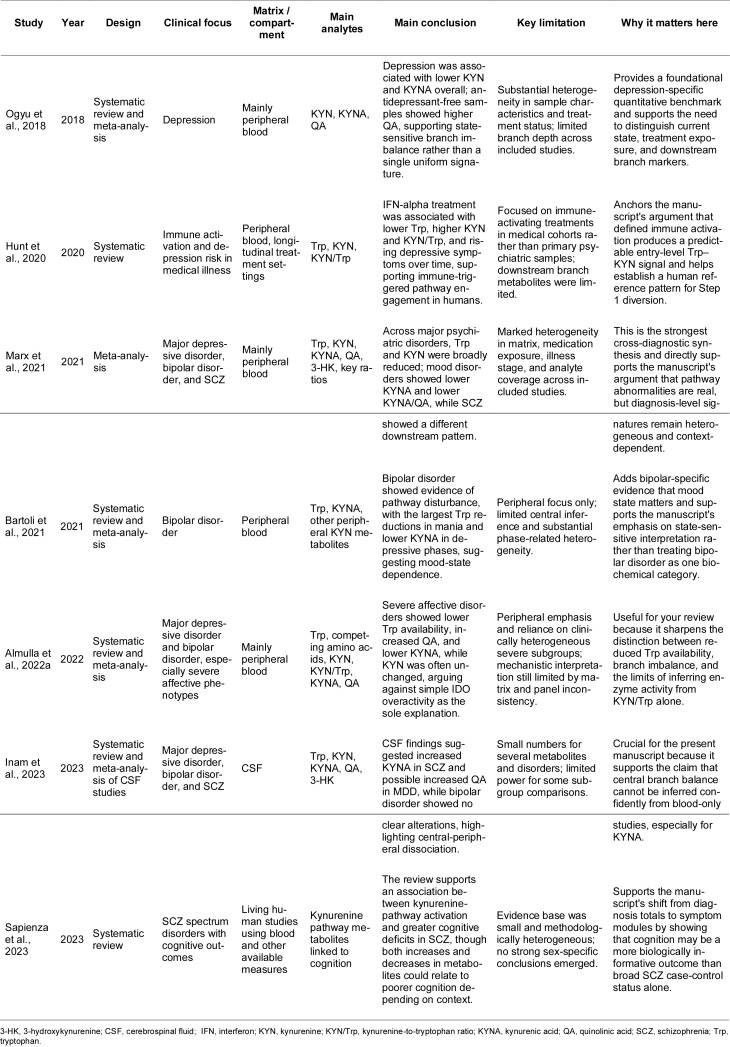
Prior meta-analyses and systematic reviews informing the current understanding of Trp-KYN alterations in psychiatric disorders

**Table 2:**
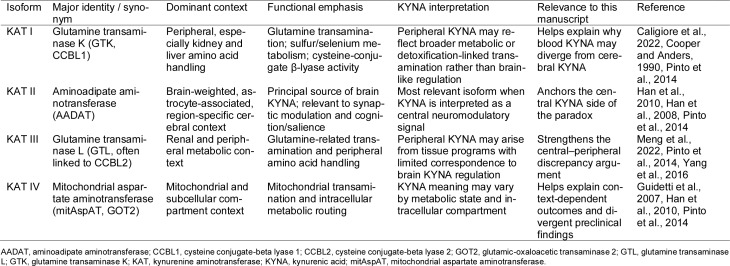
KAT isoforms as a source of interpretive heterogeneity in KYNA biology. This table emphasizes that KYNA should not be interpreted as the output of a single uniform KAT system. Differences among KAT isoforms in tissue distribution, compartmental localization, and broader metabolic roles may shape the biological meaning of KYNA across blood, brain, and other tissues. This isoform-aware framework helps explain the KYNA paradox, central-peripheral dissociation, and context-dependent KYNA-related effects.

**Table 3:**
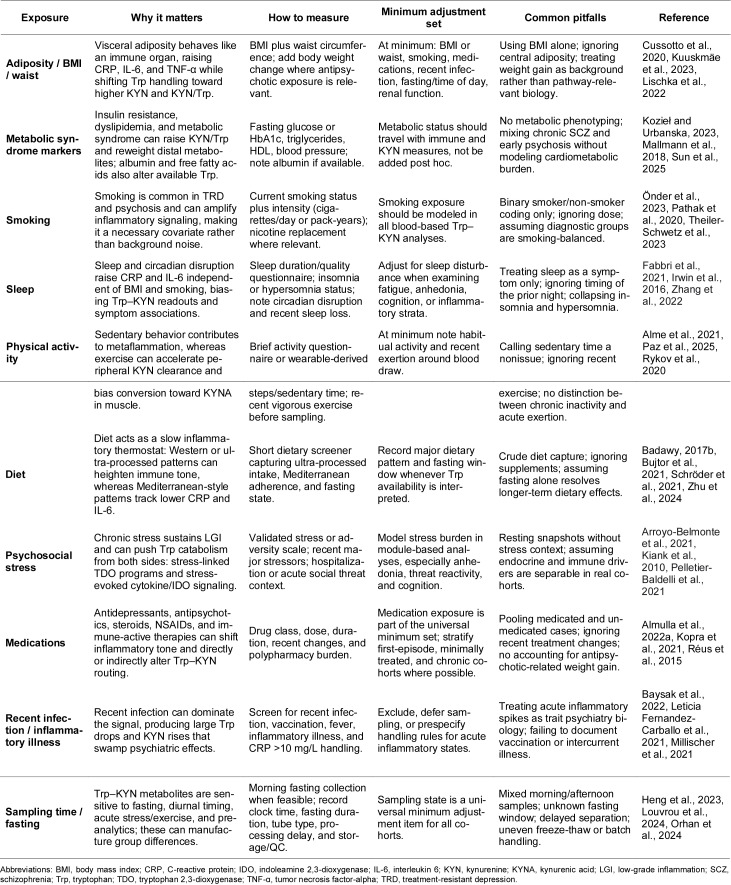
Exposure architecture that shapes inflammation-linked Trp-KYN readouts in psychiatric cohorts. This table summarizes the real-world exposures most likely to bias inflammatory tone and, in turn, distort Trp-KYN interpretation in psychiatric studies. It translates the manuscript's Sections 3.1 and 4.4 into a practical design tool by pairing each exposure with its biological rationale, a feasible measurement approach, a minimum adjustment recommendation, and the most common sources of analytic error. The aim is to make LGI less of a hidden background field and more of an explicitly modeled part of study design, stratification, and interpretation.

**Table 4:**
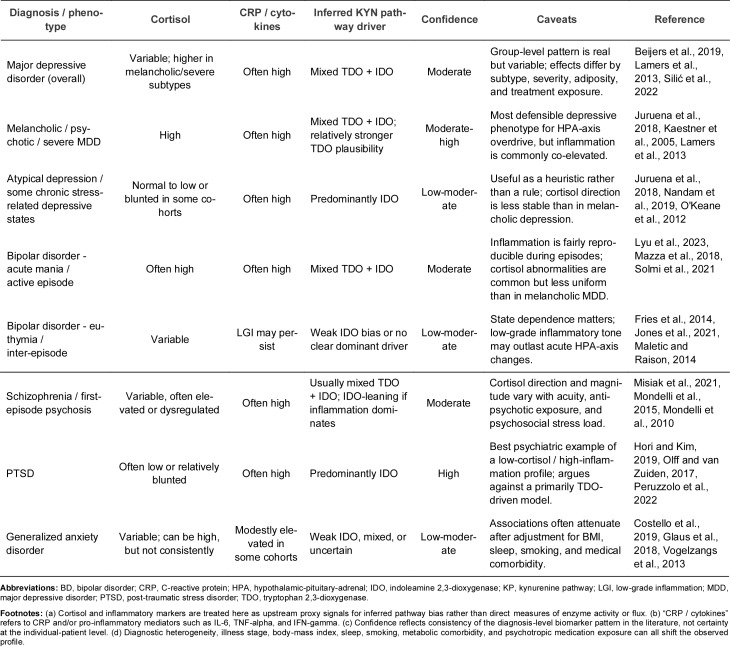
Psychiatric diagnoses stratified by cortisol and inflammatory-marker profiles, with inferred kynurenine-pathway bias. Interpretive framework: higher cortisol favors relative TDO stimulation; higher CRP and/or pro-inflammatory cytokines favor relative IDO stimulation.

**Table 5:**
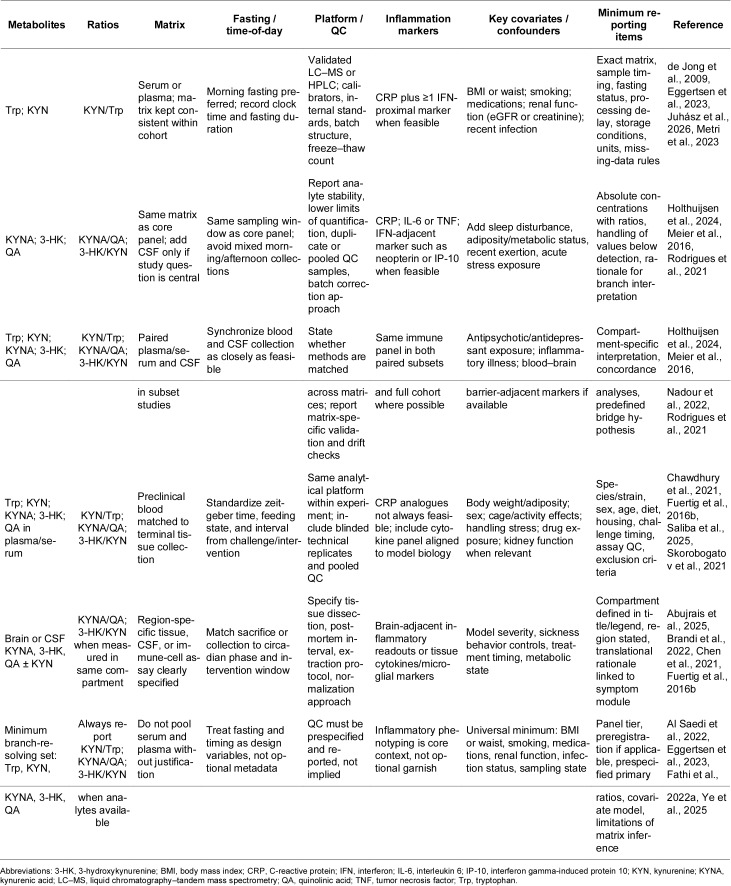
Minimal branch-resolving Trp-KYN and immune biomarker panel with reporting checklist for clinical and preclinical studies. This table operationalizes the manuscript's measurement framework by defining a minimal, branch-resolving Trp-KYN panel and the essential metadata required for interpretable psychiatric biomarker studies. It distinguishes entry-level markers from downstream branch markers, embeds immune context, and makes pre-analytic and analytical discipline explicit. The recommendations are designed for both clinical and preclinical work, with special emphasis on matrix choice, fasting and timing control, platform quality control, and dominant covariates that can distort inference, including adiposity, smoking, medications, and renal function.

**Table 6:**
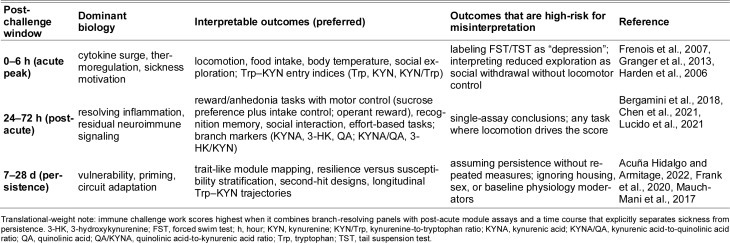
Time-sensitive interpretation of behavioral and KYN-pathway readouts after immune challenge. This table summarizes a practical time-window framework for interpreting behavioral and Trp-KYN outcomes after immune challenge. It distinguishes acute, post-acute, and persistence phases because the dominant biology, and therefore the meaning of readouts, shifts substantially across time. Early effects are driven mainly by cytokine surges, thermoregulation, and sickness motivation, whereas later windows are more informative for reward-related behavior, memory, branch-balance markers, and longer-term vulnerability phenotypes. The table also highlights high-risk interpretive errors, helping investigators avoid overcalling depression-like states, social withdrawal, or persistence when locomotor suppression, assay dependence, or missing repeated measures may better explain the findings.

**Table 7:**
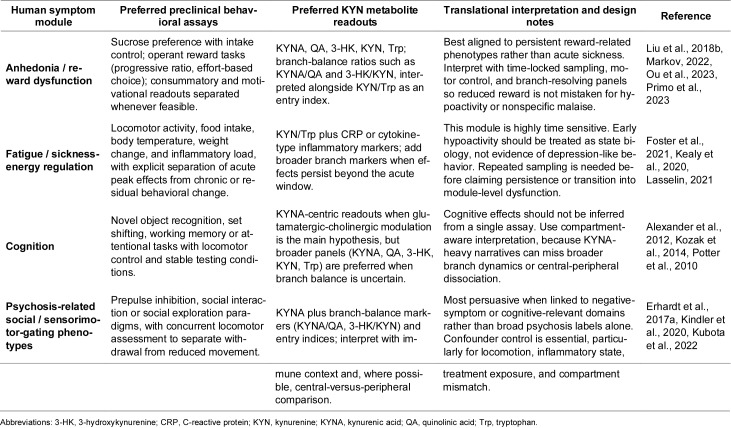
Translational alignment matrix linking human symptom modules, preclinical behavioral assays, and KYN-pathway readouts. This matrix aligns clinically meaningful symptom modules with the preclinical assays and KYN-pathway readouts most likely to preserve translational meaning across model systems. Rather than treating behavior as a generic psychiatric proxy, it organizes anhedonia, fatigue-sickness, cognition, and psychosis-related phenotypes around module-relevant tasks, branch-aware metabolite patterns, and core design safeguards. The framework also flags where interpretation becomes fragile, particularly when locomotor suppression, acute inflammatory malaise, compartment mismatch, or shallow metabolite coverage can distort inference. Used this way, the table functions as a practical bridge between human dimensional phenotyping and mechanistically interpretable preclinical Trp-KYN studies.

**Figure 1:**
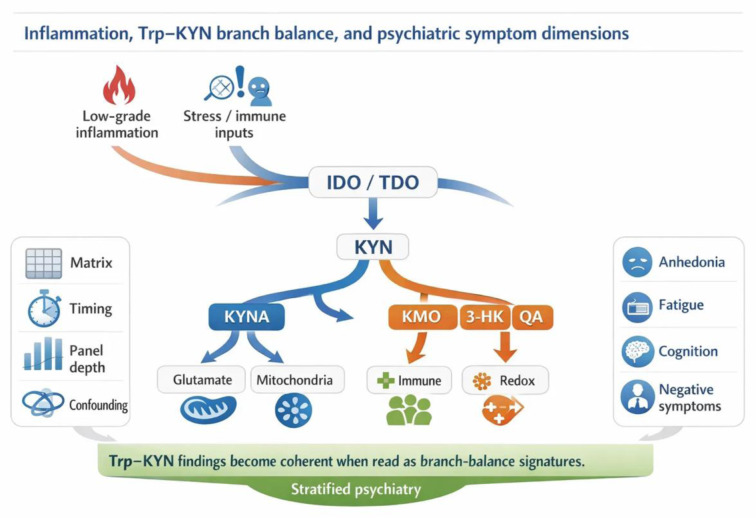
Graphical abstract
